# Characterization of the First Cultured Representative of “*Candidatus* Thermofonsia” Clade 2 within *Chloroflexi* Reveals Its Phototrophic Lifestyle

**DOI:** 10.1128/mbio.00287-22

**Published:** 2022-03-01

**Authors:** Rikuan Zheng, Ruining Cai, Chong Wang, Rui Liu, Chaomin Sun

**Affiliations:** a CAS and Shandong Province Key Laboratory of Experimental Marine Biology & Center of Deep Sea Research, Institute of Oceanology, Chinese Academy of Sciences, Qingdao, China; b Laboratory for Marine Biology and Biotechnology, Qingdao National Laboratory for Marine Science and Technology, Qingdao, China; c College of Earth Science, University of Chinese Academy of Sciences, Beijing, China; d Center of Ocean Mega-Science, Chinese Academy of Sciences, Qingdao, China; McMaster University

**Keywords:** *Chloroflexi*, “*Candidatus* Thermofonsia”, deep sea, phototrophic lifestyle, photosynthetic reaction center

## Abstract

“*Candidatus* Thermofonsia” represents a novel class within the phylum *Chloroflexi*. Metagenomic analysis reveals “*Ca.* Thermofonsia” harbors phototrophs outside the classically phototrophic *Chloroflexia* class. Unfortunately, the paucity of pure cultures limits further insights into their potential phototrophy. Here, we report the successful isolation of a “*Ca.* Thermofonsia” representative (*Phototrophicus methaneseepsis* ZRK33) from a deep-sea cold seep. Using combined physiological, genomic, and transcriptomic methods, we further show the long-wavelength light (e.g., red and infrared light) could promote the growth of strain ZRK33 and upregulate the expression of genes associated with phototrophy. In particular, strain ZRK33 has a typical phototrophic lifestyle under both laboratory and deep-sea conditions. Strain ZRK33 also possesses the ability to fix inorganic carbon through the 3-hydroxypropionate bicycle in both laboratory and deep-sea *in situ* environments, and the combined autotrophic, phototrophic, and heterotrophic capabilities endow strain ZRK33 with a photomixotrophic lifestyle. Notably, the predicted genes associated with phototrophy broadly exist in the metagenomes of 27 deep-sea *Chloroflexi* members, strongly suggesting diverse phototrophic *Chloroflexi* members are distributed in various unexplored deep biospheres.

## INTRODUCTION

Light is a rich and variable source of energy that supports life on Earth (1). Light is ubiquitously distributed in diverse environments, including the deep ocean (e.g., geothermal light and bioluminescence) (2, 3). Accordingly, phototrophic and nonphototrophic microbes have evolved different sophisticated light utilization systems to harness light for survival through photosynthetic apparatuses (4) and photosensors (5), respectively. There are two types of bacterial photosynthesis: oxygenic (cyanobacteria) and anoxygenic (sulfur and nonsulfur phototrophs) (4). Since the first observation of anoxygenic photosynthesis, eight bacterial phyla (including *Cyanobacteria*, *Chloroflexi*, *Proteobacteria*, *Firmicutes*, *Chlorobi*, *Acidobacteria*, *Gemmatimonadetes*, and “*Candidatus* Eremiobacterota”) have been reported to conduct an anoxygenic phototrophic lifestyle (6).

Among these eight photosynthetic bacterial phyla, the *Chloroflexi* (formerly called green nonsulfur bacteria) are a kind of filamentous bacteria possessing high abundance in various environments and a wide diversity of metabolisms, and their ecological roles are best known as the photoheterotrophic lifestyle (7). The well-characterized phototrophic isolates of *Chloroflexi* are within a single class, *Chloroflexia*, including the genera *Roseiflexus*, *Oscillochloris*, and *Chloroflexus* (8). Phototrophic *Chloroflexi* could synthesize bacteriochlorophylls or other pigments to capture light (9). Unlike phototrophic isolates of *Chlorobi*, *Heliobacteria*, and *Acidobacteria* bacteria, which possess a type I photosystem (RCI) (10), phototrophic members of *Chloroflexi*, *Proteobacteria*, and *Gemmatimonadetes* utilize a type II photosystem (RCII) (11, 12). In addition, photoautotrophic *Chloroflexi* fix CO_2_ using either the 3-hydroxypropionate (3HP) pathway (13, 14) or the reductive pentose phosphate cycle (15). For example, three thermophilic phototrophic *Chloroflexi* bacteria (*Roseiflexus* sp. strain RS-1, Roseiflexus castenholzii, and Chloroflexus aggregans) can fix CO_2_ using the 3HP pathway (16). In contrast, Oscillochloris trichoides DG-6, a photosynthetic *Chloroflexi* strain isolated from a warm hydrogen sulfide spring, uses the reductive pentose phosphate pathway to fix carbon (17).

At the time of writing, the phylum *Chloroflexi* is phylogenetically divided into nine classes, including *Chloroflexia* (18), *Anaerolineae* (19), *Caldilineae* (19), *Ktedonobacteria* (20), *Thermomicrobia* (21), *Dehalococcoidia* (22), *Tepidiformia* (23), *Thermoflexia* (24), and *Ardenticatenia* (25). In addition, there are some unclassified groups, such as SAR202 clade (26), “*Ca.* Thermofonsia” (9), and “*Candidatus* Limnocylindria” (27). Besides the phototrophic *Chloroflexia* class, accumulating evidence suggests phototrophy also is present in other classes, and the diversity of phototrophy in the *Chloroflexi* phylum is richer than previously thought (9). In particular, the recent description of “*Ca.* Thermofonsia” (9), a new class-level clade, contains members with diverse metabolic pathways that distinguished it from its sister class, *Anaerolineae* (9). Notably, “*Ca.* Thermofonsia” clade 2 encompasses members with the potential for reaction center-based phototrophy, and these members are proposed to represent a novel lineage of anoxygenic phototrophy in *Chloroflexi* outside the well-characterized *Chloroflexia* class (9). However, this hypothesis is only based on metagenomics analysis, and a pure culture of “*Ca.* Thermofonsia” is needed for better understanding and verifying their predicted phototrophic lifestyle.

Here, we cultured the first representative (named *Phototrophicus methaneseepsis* ZRK33) of “*Ca.* Thermofonsia” clade 2 from the deep-sea sediment. Combining physiological and transcriptomic approaches, we confirmed the phototrophic lifestyle of this new isolate under both laboratory and deep-sea conditions. We found strain ZRK33 could perform the 3HP bicycle in both laboratory light illumination and *in situ* light. Lastly, the broad distribution of phototrophic genes in deep-sea *Chloroflexi* bacteria was also revealed.

## RESULTS

### The relative abundance of deep-sea *Chloroflexi* bacteria.

To gain preliminary insights into *Chloroflexi* bacteria existing in deep-sea environments, operational taxonomic unit (OTU) sequencing was first performed to detect the relative abundance in small subunit (SSU) rRNA gene tag sequencing of the phylum *Chloroflexi* present in the cold seep and hydrothermal vent sediments. The result showed that the *Chloroflexi* group was the second most abundant phylum in both cold seep and hydrothermal vents, suggesting *Chloroflexi* bacteria were dominant in these environments (see Fig. S1A and S1B in the supplemental material). The proportion of *Chloroflexi* accounted for 6.15%, 10.92%, 5.04%, 8.67%, and 13.67% of the whole bacterial domain at the phylum level in samples RPC, ZC1, ZC2, H1, and H2 (Fig. S1A and B). At the class level, *Dehalococcoidia* and *Anaerolineae* were the top two classes in both cold seep (Fig. S1C) and hydrothermal vent sediments (Fig. S1D).

10.1128/mbio.00287-22.2 FIG S1 Detection of the abundance of the phylum *Chloroflexi* derived from deep-sea cold seep and hydrothermal vent sediments. Download FIG S1, DOCX file, 0.2 MB. Copyright © 2022 Zheng et al. 2022 Zheng et al. https://creativecommons.org/licenses/by/4.0/ This content is distributed under the terms of the Creative Commons Attribution 4.0 International license.

### Cultivation and morphology of a novel deep-sea *Chloroflexi* strain.

**FIG 1:**
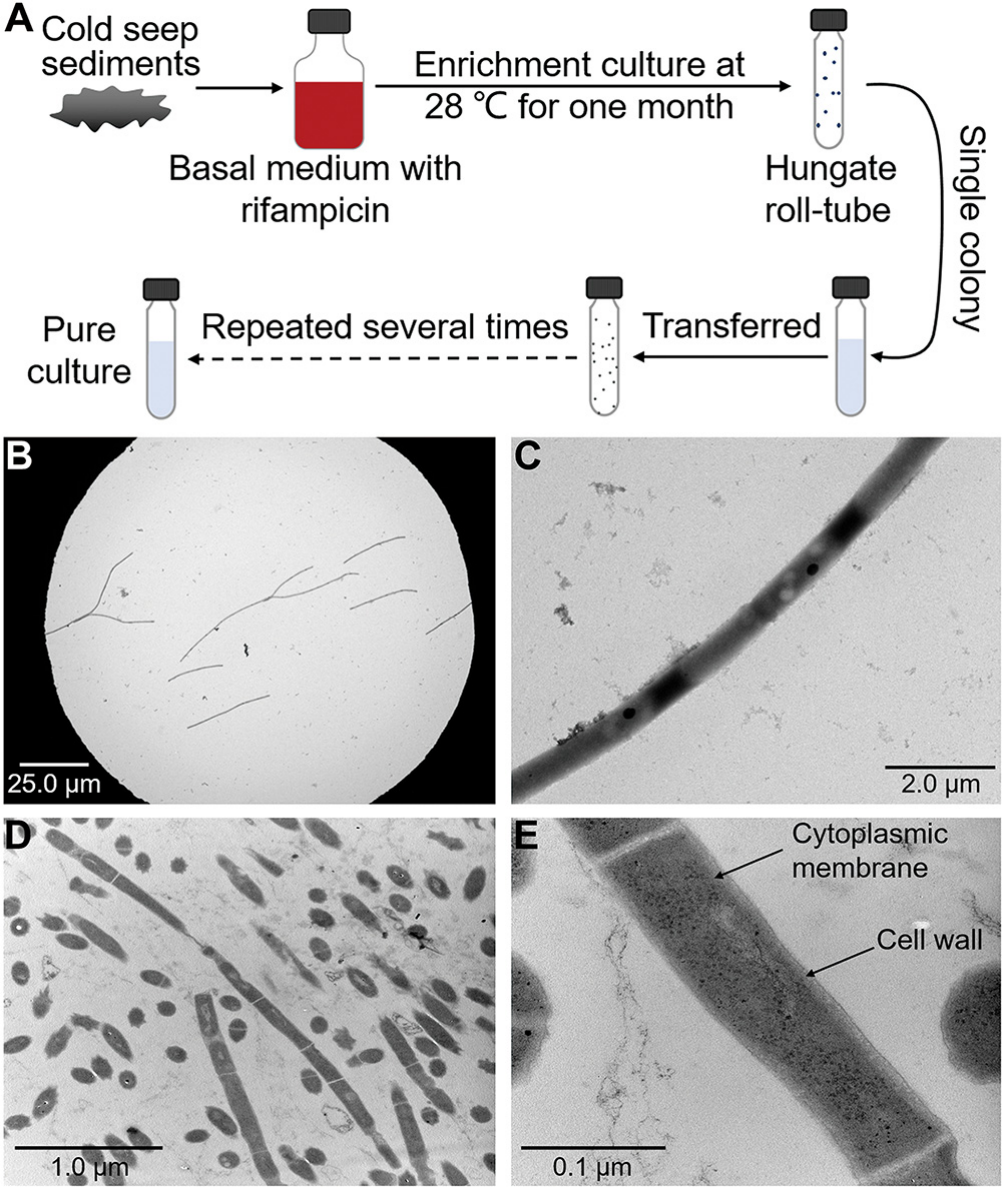
Isolation and morphology of the *Chloroflexi* strain ZRK33. (A) Diagrammatic scheme of enrichment and isolation of *Chloroflexi* bacteria from the deep-sea cold seep samples. (B and C) TEM observation of strain ZRK33. (D and E) TEM observation of the ultrathin sections of strain ZRK33.

To isolate *Chloroflexi* bacteria from deep-sea sediment samples RPC, ZC1, ZC2, H1, and H2, we developed an enrichment strategy by using a basal medium constantly supplemented with 50 μg/mL rifampicin, given that some members of *Chloroflexi* were reported to tolerate rifampicin (28, 29) and a lot of bacteria were sensitive to this antibiotic. Using this strategy, we anaerobically enriched these deep-sea sediment samples at 28°C for 1 month. Thereafter, the enrichments were plated on the solid medium in Hungate tubes, and individual colonies with distinct morphology were picked and cultured (Fig. 1A). Some of the cultured colonies from samples ZC1 and H2 were identified as *Chloroflexi* bacteria based on their 16S rRNA gene sequences. Among them, strain ZRK33 possessed a high growth rate and was chosen for further study. Under TEM observation, the cells of strain ZRK33 were filamentous, with a length of more than 20 μm and a width of 0.5 to 0.6 μm, and had no flagellum (Fig. 1B and C). Ultrathin sections of whole cells of strain ZRK33 revealed a cytoplasmic membrane surrounded by a cell wall surface layer (Fig. 1D and E). The strain did not possess a clearly visible sheath-like structure (Fig. 1E) like that shown in Pelolinea submarina MO-CFX1, a typical *Chloroflexi* bacterium belonging to the class *Anaerolineae* (29).

### Physiological characteristics, genome, and phylogeny of strain ZRK33.

The detailed physiological characteristics of strain ZRK33 and other *Chloroflexi* bacteria are listed in Table S1. The optimum temperature for growth of strain ZRK33 was 28°C, similar to strains MO-CFX2 and MO-CFX1, while apparently different from strains UNI-1, IMO-1, and P3M-1. The cell morphology, optimum pH for growth, and NaCl concentration for growth of strain ZRK33 were similar to those of other *Chloroflexi* bacteria. The most abundant fatty acids derived from strain ZRK33 were different from those from phylogenetically close relatives (Table S1). To assess genomic features of strain ZRK33, its whole genome was sequenced and analyzed (genome accession number CP051151). The number of contigs was 1, and the genome size of strain ZRK33 was 5,631,885 bp with a DNA G+C content of 52.76% (Fig. S2A). Annotation of the genome of strain ZRK33 revealed it consisted of 4,885 predicted genes, including 55 RNA genes (6 rRNA genes, 46 tRNA genes, and 3 other ncRNAs). To further clarify the phylogenetic position of strain ZRK33, the genome relatedness values were calculated by the average nucleotide identity (ANI), *in silico* DNA-DNA similarity (*is*DDH), and tetranucleotide signatures (Tetra) against six genomes (strain ZRK33 and five strains, MO-CFX2, MO-CFX1, UNI-1, IMO-1, and P3M-1, belonging to class *Anaerolineae*) (Table S1). The ANIs based on the BLASTN algorithm (ANIb) of ZRK33 with strains MO-CFX2, MO-CFX1, UNI-1, IMO-1, and P3M-1 were 64.81%, 63.06%, 63.42%, 63.41%, and 63.29%, respectively. The ANIs based on the MUMMER ultrarapid aligning tool (ANIm) of ZRK33 with MO-CFX2, MO-CFX1, UNI-1, IMO-1, and P3M-1 were 85.21%, 82.63%, 83.42%, 83.15%, and 83.23%, respectively. The Tetra values of ZRK33 with MO-CFX2, MO-CFX1, UNI-1, IMO-1, and P3M-1 were 0.48145, 0.67572, 0.64677, 0.65234, and 0.65126, respectively. Based on digital DNA-DNA hybridization employing the Genome-to-Genome Distance Calculator (GGDC), the *in silico* DDH estimates for ZRK33 with MO-CFX2, MO-CFX1, UNI-1, IMO-1, and P3M-1 were 23.30%, 24.20%, 20.40%, 21.60%, and 23.80%, respectively. These results together indicated the genome of strain ZRK33 is obviously below established cutoff values (ANIb, 95%; ANIm, 95%; *is*DDH, 70%; Tetra, 0.99) for defining bacterial species, suggesting strain ZRK33 represented a novel species within the phylum *Chloroflexi* as currently defined.

10.1128/mbio.00287-22.3 FIG S2 Genomic map and phylogenetic analysis of strain ZRK33. Download FIG S2, DOCX file, 0.8 MB. Copyright © 2022 Zheng et al. 2022 Zheng et al. https://creativecommons.org/licenses/by/4.0/ This content is distributed under the terms of the Creative Commons Attribution 4.0 International license.

10.1128/mbio.00287-22.6 TABLE S1 Characteristics of strain ZRK33 and the other isolated *Chloroflexi* members. Download Table S1, DOCX file, 0.04 MB. Copyright © 2022 Zheng et al. 2022 Zheng et al. https://creativecommons.org/licenses/by/4.0/ This content is distributed under the terms of the Creative Commons Attribution 4.0 International license.

**FIG 2:**
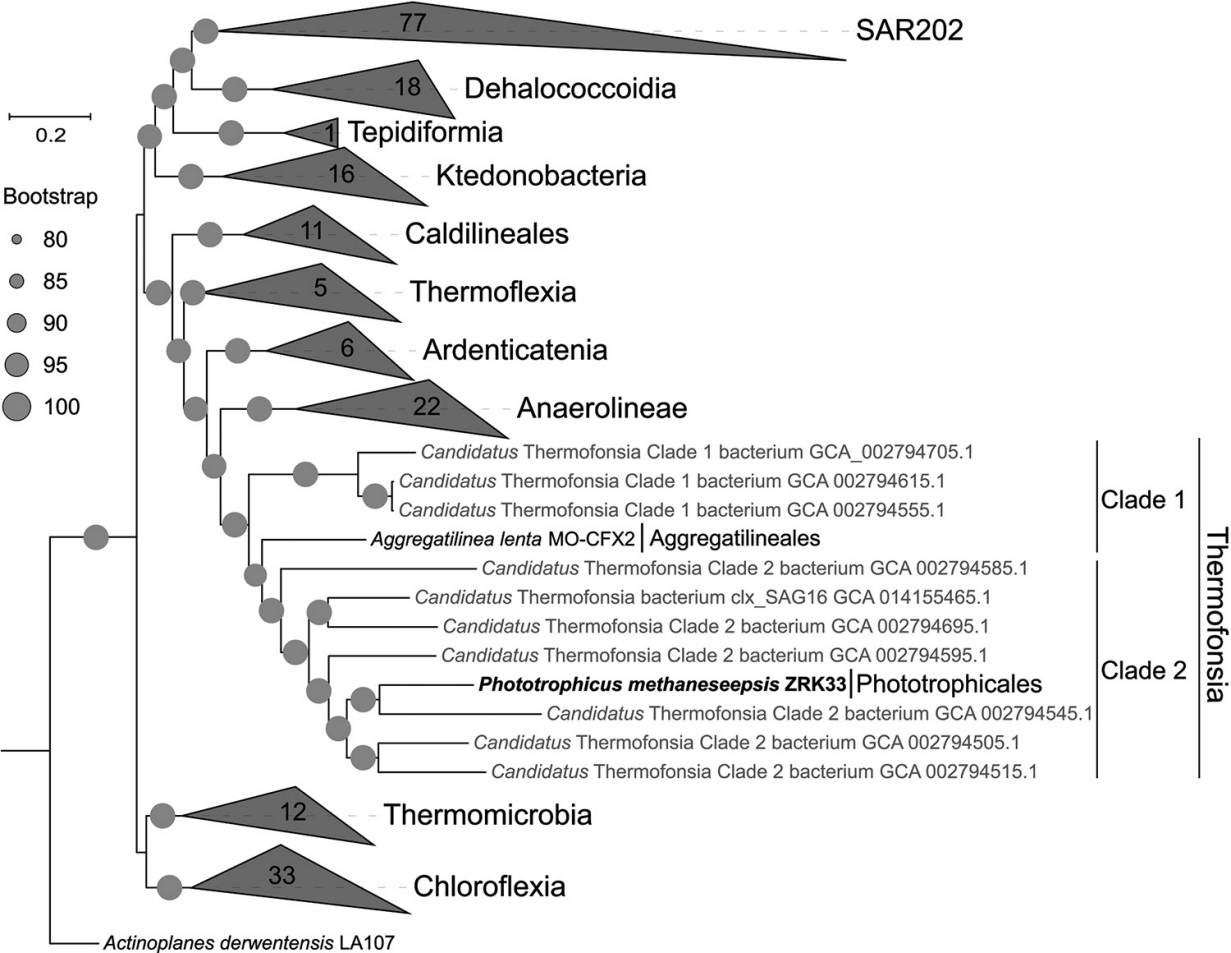
Phylogeny of the novel cultured *Chloroflexi* strain *Phototrophicus methaneseepsis* ZRK33. Maximum likelihood phylogenetic tree containing the genomes of *P*. *methaneseepsis* ZRK33 and other *Chloroflexi* representatives was constructed based on the concatenated alignment of 37 single-copy genes. The genome of Actinoplanes derwentensis LA107 is used as the outgroup. Collapsed wedges represent the monophyletic groups of genomes. Names indicated with gray color in quotation marks represent taxa that are not yet validly published. All uncultured bacterial genomes are labeled with their NCBI accession numbers. Bootstrap values of >80% are indicated at the base of each node with the gray dots (expressed as percentages of 1,000 replications). Bar, 0.2 substitutions per nucleotide position.

To further confirm the taxonomic status of strain ZRK33, we performed phylogenetic analysis with all cultured *Chloroflexi* representatives and some uncultured *Chloroflexi* members. The maximum likelihood tree of genomes placed strain ZRK33 as a new member of “*Ca.* Thermofonsia” clade 2 (Fig. 2) based on a concatenated alignment of 37 protein-coding genes. Consistent with this, the maximum likelihood trees of the 16S rRNA gene sequence (Fig. S2B) further confirmed that strains ZRK33 and MO-CFX2 together formed a monophyletic clade sister to the *Anaerolineae* class. Interestingly, strain MO-CFX2 was previously considered to represent a novel order within the class *Anaerolineae* (28). A recent metagenomics-based analysis proposed the reassignment of “*Ca.* Thermofonsia” into the class *Anaerolineae* (30). Our phylogenetic analyses clearly revealed that “*Ca.* Thermofonsia” should be a novel class separated from all other reported classes. Correspondingly, strain ZRK33, strain MO-CFX2, and all other uncultured “*Ca.* Thermofonsia” members were proposed to form a novel class within the phylum *Chloroflexi* (Fig. 2). Strain ZRK33 possesses a clear classification status within the proposed class “*Ca.* Thermofonsia,” which is qualified to be named *Thermofonsia* here.

In addition, based on the 16S rRNA gene sequence of strain ZRK33, a sequence similarity calculation using the NCBI server indicated that the closest relative of strain ZRK33 within *Thermofonsia* was *Aggregatilinea lenta* MCOF-2 (82.81%; order *Aggregatilineales*). Recently, the proposed minimum threshold of 16S rRNA sequence identity value for a new order has been revealed as 83.55% (31). Together, based on phylogenetic, genomic, and phenotypic characteristics, we proposed that strain ZRK33 was classified as a representative of a novel order of the class *Thermofonsia*. Given the broad distribution of genes associated with anoxygenic photosynthesis in the strain ZRK33 genome and its phototrophic lifestyle, as shown in this study, we propose the name *Phototrophicus methaneseepsis* gen. nov., sp. nov., for strain ZRK33. In addition, we also propose the associated family and order as *Phototrophicaceae* fam. nov. and *Phototrophicales* ord. nov., respectively.

### Description of *Phototrophicus* gen. nov. and *Phototrophicus methaneseepsis* sp. nov.

*Phototrophicus* (Pho.to'tro.phi.cus. L. fem. n. *Photo*, photosynthesis; L. adj. *trophic* trophic modes; N.L. fem. n. *Phototrophicus* phototrophic organism).

Facultatively anaerobic, mesophilic, neutrophilic and moderately halophilic (Table S1). Cells are nonmotile. Gram-staining reaction is negative. The phylogenetic position is in the family *Phototrophicaceae*, order *Phototrophicales* within the class *Thermofonsia* of the phylum *Chloroflexi*. The type species is *Phototrophicus methaneseepsis*.

*Phototrophicus methaneseepsis* (me.th.ane'seep.sis. L. gen. pl. n. *methaneseepsis* of the deep-sea methane seeps). Cells are generally more than 20 μm long and 0.5 to 0.6 μm wide, filamentous, facultatively anaerobic, and have no flagellum. The sole carbon source utilization test showed that the growth of strain ZRK33 is stimulated by arabinose, fructose, glucose, galactose, mannose, ribose, fumarate, pyruvate, and peptone. Growing at pH 6.0 to 8.0 (optimum, pH 7.0). The temperature range for growth is 4 to 37°C with an optimum at 28°C. Growth occurs at NaCl concentrations between 0.0 and 5.0% with optimum growth at 3.0% NaCl. Containing significant proportions (>10%) of the cellular fatty acids C_16:0_, C_15:0_2-OH, C_17:1_*ω*6c, and C_18:1_*ω*7c. The type strain, ZRK33^T^, was isolated from the sediment of a deep-sea cold seep near the People’s Republic of China. The DNA G+C content of the type strain is 52.76%.

The detailed descriptions of other levels of family *Phototrophicaceae* and order *Phototrophicales* are shown Text S1.

10.1128/mbio.00287-22.1 TEXT S1 Detailed procedures of transcriptomic sequencing analysis and real-time quantitative reverse transcription PCR. Detailed description of *Phototrophicaceae* fam. nov. and *Phototrophicales* ord. nov. is given. Download Text S1, DOCX file, 0.03 MB. Copyright © 2022 Zheng et al. 2022 Zheng et al. https://creativecommons.org/licenses/by/4.0/ This content is distributed under the terms of the Creative Commons Attribution 4.0 International license.

### Genomic and physiologic evidences associated with the phototrophic lifestyle of *P*. *methaneseepsis* ZRK33.

As previously reported (9), the draft genomes of some uncultured members of the class *Thermofonsia* clade 2 contained many genes closely associated with anoxygenic phototrophy, and *Thermofonsia* clade 2 was speculated to be a novel phototrophic bacterial lineage belonging to the *Chloroflexi* phylum (9). Given that strain ZRK33 was proposed to be a representative of *Thermofonsia* clade 2, we analyzed the genome of strain ZRK33 to explore the genetic signatures associated with phototrophy. Strain ZRK33 possessed many genes encoding essential apparatuses involved in anoxygenic phototrophy, including the type II reaction center, bacteriochlorophyll synthase, cytochrome *b*_6_/*f* complex, photosynthetic electron transport system, F-type ATPase, and 3HP bicycle but no alternative complex III (Table S2). The existence of these genes strongly suggested that strain ZRK33 has a phototrophic lifestyle. Until now, there were nine classes within the phylum *Chloroflexi* identified based on the cultured representative strains (Table S3), and only one class (*Chloroflexia*) was experimentally demonstrated as a phototrophic lineage (9). Given the special taxonomic positions of strain ZRK33 as well as *Thermofonsia* in phylogenetic trees, strain ZRK33 might represent a novel type of phototrophic bacteria within the phylum *Chloroflexi.*

10.1128/mbio.00287-22.7 TABLE S2 Phototrophy-associated genes in the genome of strain ZRK33. Download Table S2, DOCX file, 0.02 MB. Copyright © 2022 Zheng et al. 2022 Zheng et al. https://creativecommons.org/licenses/by/4.0/ This content is distributed under the terms of the Creative Commons Attribution 4.0 International license.

10.1128/mbio.00287-22.8 TABLE S3 Characteristics of the new class *Thermofonsia* (with strain ZRK33 as a representative) and other *Chloroflexi* classes. Download Table S3, DOCX file, 0.04 MB. Copyright © 2022 Zheng et al. 2022 Zheng et al. https://creativecommons.org/licenses/by/4.0/ This content is distributed under the terms of the Creative Commons Attribution 4.0 International license.

**FIG 3:**
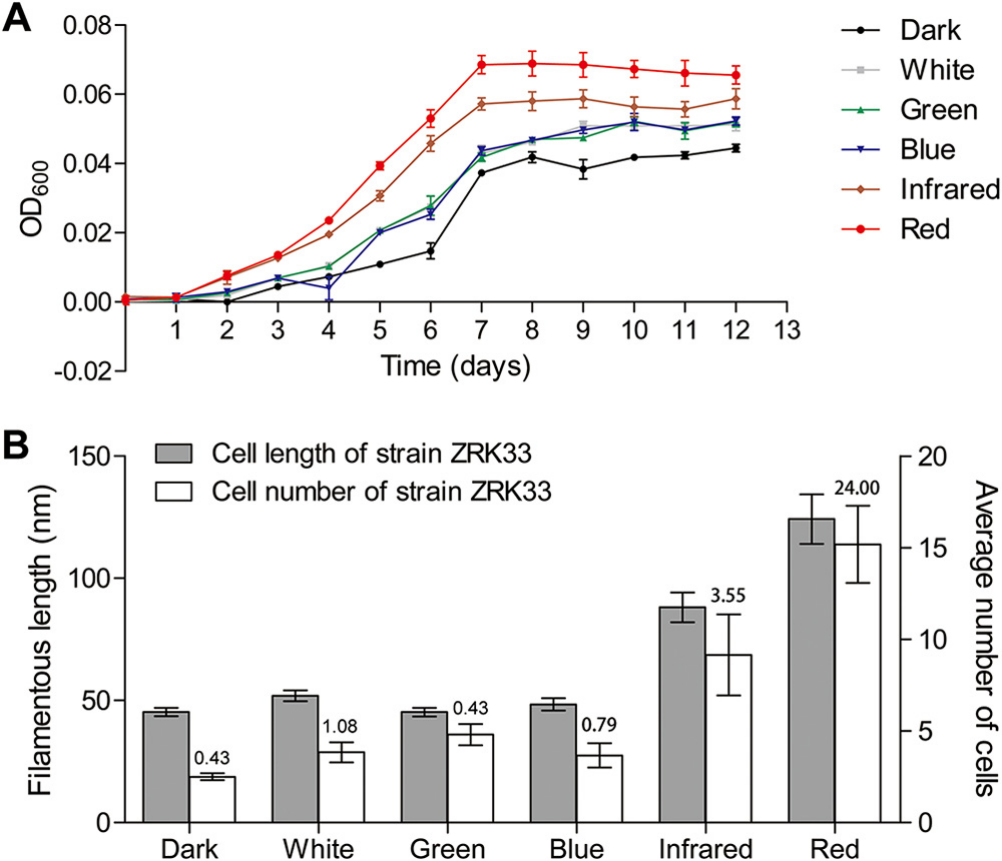
Light promotes the growth of *P*. *methaneseepsis* ZRK33. (A) Growth assay of strain ZRK33 cultivated in rich medium exposed to darkness and different wavelengths of light illumination. (B) Effects of different light exposures on the filamentous cell length and number of strain ZRK33. The average cell length and number of strain ZRK33 are calculated based on 50 photographs taken randomly for each condition. The ratios of numbers of long (>50 μm) to short (<50 μm) filament cells of strain ZRK33 cultured under darkness and different wavelengths of light illumination are shown at the top of the bar chart.

Next, we sought to explore the effect of illumination on the growth of strain ZRK33. With that, strain ZRK33 was cultured anaerobically under darkness and different wavelengths of light. The illumination could promote the growth rate to some extent compared to the dark condition, and the long-wavelength light (including red and infrared light) was revealed as the best illumination source to facilitate the growth of strain ZRK33 (Fig. 3A). Consistent with this, the number of filament cells of strain ZRK33 cultured under the exposure of red (with an average number of about 15.2) or infrared (with an average number of about 9.1) light was evidently higher than those under other illumination conditions (e.g., with an average number of about 3.8, 5.0, and 3.7 under exposure of white, green, and blue light, respectively) and darkness (with an average number of about 2.5) (Fig. 3B and Fig. S3). In parallel, the average length of filament cells of strain ZRK33 cultured under the exposure of red (∼124.2 μm) or infrared (∼88.1 μm) light was much longer than those under other illumination conditions (white, ∼51.9 μm; green, ∼45.2 μm; blue, ∼48.4 μm) and darkness (∼45.3 μm) (Fig. 3B). In addition, the ratio of numbers of long (>50 μm) to short (<50 μm) filament cells of strain ZRK33 cultured under the exposure of red (∼24.00) or infrared light (∼3.55) was higher than those under other illumination conditions (e.g., white, ∼1.08; green, ∼0.43; blue, ∼0.79) and darkness (∼0.43) (Fig. 3B). Overall, these results highlight that strain ZRK33 is capable of utilizing light, especially the long-wavelength light, as an energy source to promote its growth.

10.1128/mbio.00287-22.4 FIG S3 Representative pictures showing the number and length of filamentous cells of strain ZRK33 under dark (A) and different wavelengths of light illumination (including white [B], green [C], blue [D], infrared [E] and red [F]). Download FIG S3, DOCX file, 0.4 MB. Copyright © 2022 Zheng et al. 2022 Zheng et al. https://creativecommons.org/licenses/by/4.0/ This content is distributed under the terms of the Creative Commons Attribution 4.0 International license.

### Transcriptomic assays of the involvement of phototrophy-associated genes in the light utilization of *P*. *methaneseepsis* ZRK33 cultured under both laboratory and deep-sea conditions.

**FIG 4:**
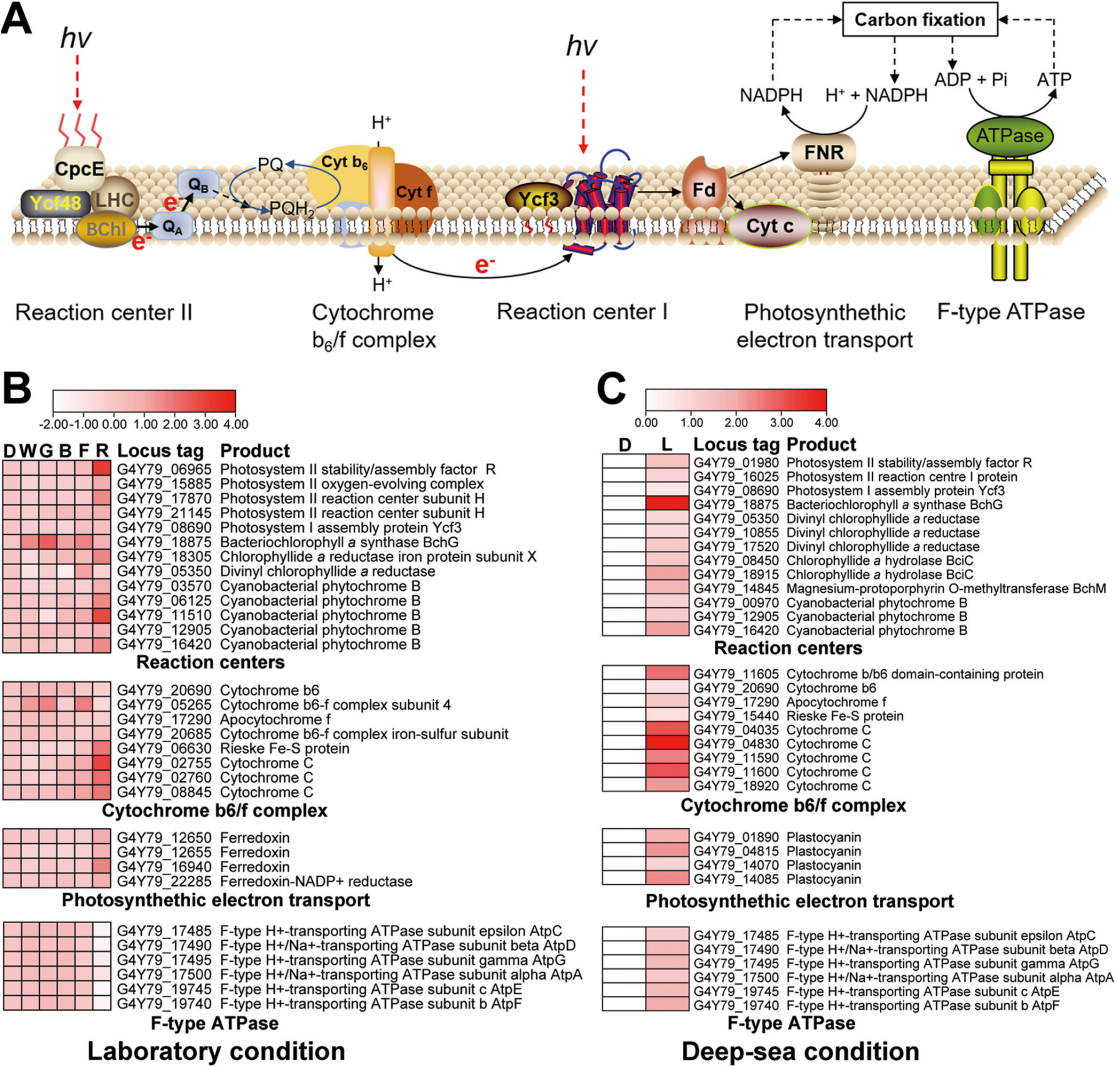
Transcriptomic analysis of the phototrophic lifestyle conducted by *P*. *methaneseepsis* ZRK33 under both laboratory and deep-sea conditions. (A) Schematic representation of the components of a typical phototrophic system identified in the photosynthetic bacteria. (B) Transcriptomics-based heat map showing the differentially expressed genes associated with phototrophic apparatus of *P*. *methaneseepsis* ZRK33 exposed to darkness and different wavelengths of light under laboratory conditions. D, dark; W, white; G, green; B, blue; I, infrared; R, red. (C) Transcriptomics-based heat map showing all upregulated genes associated with phototrophic apparatus of *P*. *methaneseepsis* ZRK33 with a 10-day incubation exposed to the natural light of a deep-sea cold seep. D, dark; L, light.

It is well known that phototrophic apparatuses (including reaction center, cytochrome *b*_6_/*f* complex, photosynthetic electron transport, and ATPase) are crucial for a typical phototrophic bacterium to capture light and generate energy (Fig. 4A). Given that there is a complete set of phototrophy-associated genes existing in the genome of strain ZRK33 (Table S2) and light could stimulate the growth of strain ZRK33 (Fig. 3), we next sought to ask whether these identified genes indeed contributed to the light utilization of strain ZRK33. To this end, we performed the transcriptomic analysis of strain ZRK33 that was cultured under darkness and different wavelengths of light. The transcriptomic results revealed that the expression of genes associated with the reaction center, cytochrome *b*_6_/*f* complex, and photosynthetic electron transport system were significantly upregulated under red light and slightly upregulated under infrared light compared to other light and darkness conditions (Fig. 4B), consistent with quantitative reverse transcription-PCR (qRT-PCR) results (Fig. S4A). In particular, the expression of the *bchG* gene (encoding bacteriochlorophyll *a* synthase) was upregulated simultaneously under different lights (white, green, blue, infrared, and red), indicating its key role in the light utilization of strain ZRK33 (Fig. 4B). However, unexpectedly, transcripts for genes encoding F-type ATPase were all downregulated under infrared and red light compared to other light irradiations (Fig. 4B), suggesting strain ZRK33 generates ATP through other types of ATPase when exposed to the long-wavelength light.

10.1128/mbio.00287-22.5 FIG S4 qRT-PCR detection of expression changes of genes associated with phototrophic apparatus and 3HP bicycle of strain ZRK33 under laboratory and deep-sea conditions. Download FIG S4, DOCX file, 0.2 MB. Copyright © 2022 Zheng et al. 2022 Zheng et al. https://creativecommons.org/licenses/by/4.0/ This content is distributed under the terms of the Creative Commons Attribution 4.0 International license.

Considering strain ZRK33 was isolated from the deep-sea environment, we next sought to explore the response of strain ZRK33 to the natural light existing in the deep ocean. Thus, we performed the *in situ* cultivation of strain ZRK33 in anaerobic bags either without or with exposure to the outside illuminant environment for 10 days. The cells were collected and we performed transcriptomic analyses. Interestingly, the transcriptomic results clearly showed that the expression of many genes encoding essential factors associated with reaction center, cytochrome *b*_6_/*f* complex, photosynthetic electron transport system, and F-type ATPase were significantly upregulated compared to that cultured under dark conditions (Fig. 4C), consistent with qRT-PCR results (Fig. S4C). Notably, the expression of genes encoding various components of F-type ATPase was evidently upregulated, suggesting a mixed form of light exists in the cold seeps. Overall, strain ZRK33 was capable of utilizing light as an energy source for growth under both laboratory and deep-sea conditions, and phototrophy-associated genes existing in the genome were involved in the process of light utilization.

### *P*. *methaneseepsis* ZRK33 possesses the ability to fix inorganic carbon through the 3HP bicycle under light.

**FIG 5:**
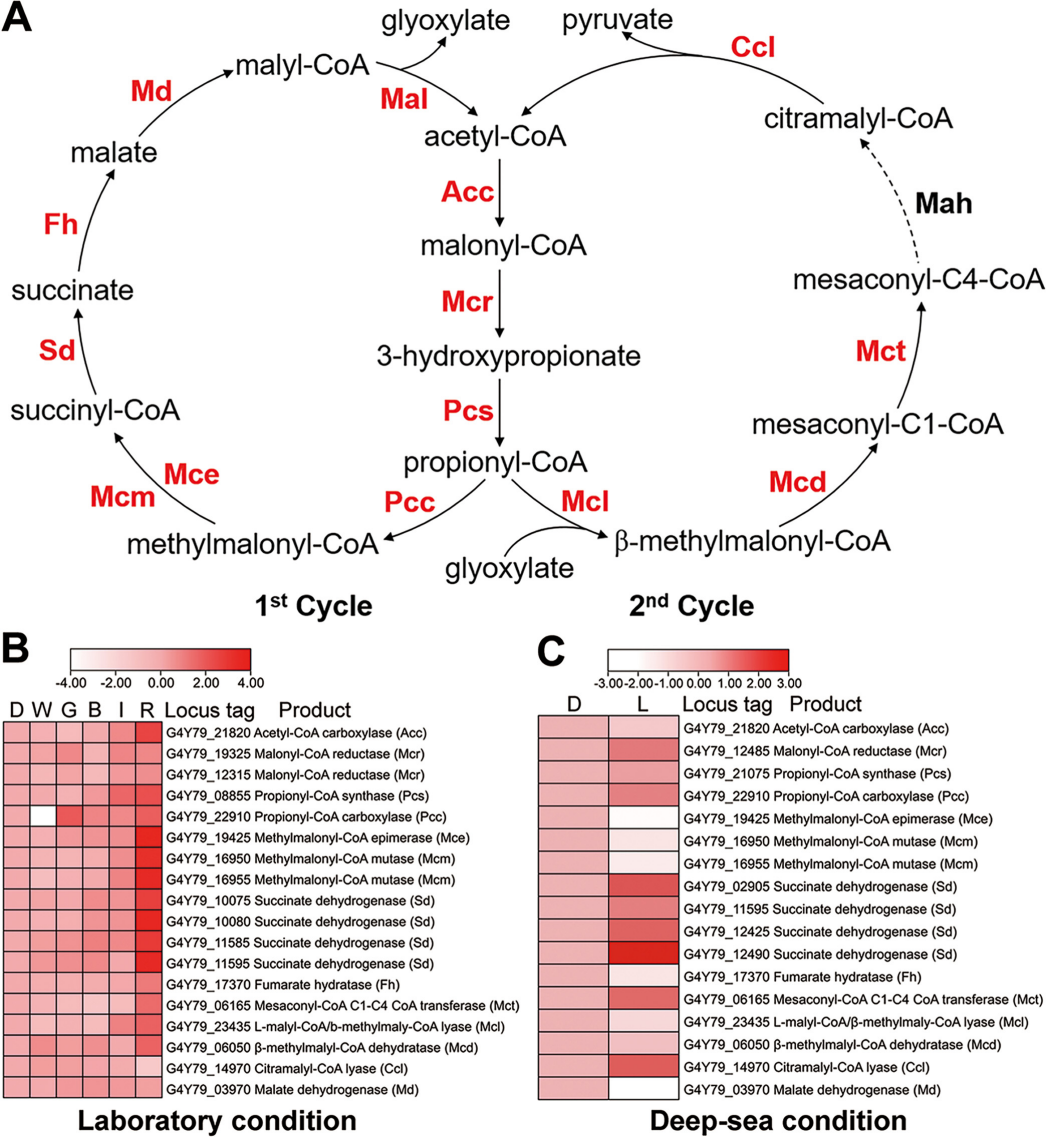
*P*. *methaneseepsis* ZRK33 has the ability to fix inorganic carbon under the illumination condition via the 3HP bicycle. (A) A metabolic schematic illustrating the 3HP bicycle identified in strain ZRK33. Enzymes involved in the pathway are highlighted by red color, except the one absent from strain ZRK33 is black. Acc, acetyl-CoA carboxylase; Mcr, malonyl-CoA reductase; Pcs, propionyl-CoA synthase; Pcc, propionyl-CoA carboxylase; Mce, methylmalonyl-CoA epimerase; Mcm, methylmalonyl-CoA mutase; Sd, succinate dehydrogenase; Fh, fumarate hydratase; Mct, succinyl-CoA–l-malate-CoA transferase; Mcl, l-malyl-CoA–β-methylmaly-CoA lyase; Mcd, β-methylmalyl-CoA dehydratase; Ccl, citramalyl-CoA lyase; Md, malate dehydrogenase. (B) Transcriptomics-based heat map showing the differentially expressed genes associated with 3HP bicycle of strain ZRK33 exposed to darkness and different wavelengths of light illumination under the laboratory condition. D, dark; W, white; G, green; B, blue; I, infrared; R, red. (C) Transcriptomics-based heat map showing the differentially expressed genes associated with the 3HP bicycle of strain ZRK33 with a 10-day incubation exposed to the natural light of the deep-sea cold seep. D, dark; L, light.

It has been reported that the typical photosynthetic *Chloroflexi* bacterium Chloroflexus aurantiacus was facultative autotrophic (14), and it had the ability to fix inorganic carbon in light via the 3HP bicycle. Based on the genomic analysis, nearly all genes associated with the 3HP bicycle (with the exception of the gene encoding mesaconyl-C4-coenzyme A [CoA] hydratase) were found in the genome of strain ZRK33 (Table S2 and Fig. 5A). We speculated strain ZRK33 can fix carbon via the 3HP bicycle. We next sought to explore the occurrence of the 3HP bicycle in the light through the transcriptomic approach. Indeed, when exposed to different wavelengths of light in the laboratory, the expression of almost all genes involved in the 3HP bicycle was upregulated (Fig. 5B), verified via qRT-PCR analyses (Fig. S4B). In particular, the process of the 3HP bicycle was significantly upregulated under red light stimulation compared to other wavelengths of light (Fig. 5B), consistent with the growth promotion of strain ZRK33 (Fig. 3A) and expression upregulation of phototrophy-associated genes (Fig. 4B) by red light. Moreover, all the transcriptomic and qRT-PCR results based on the *in situ* cultured bacteria showed the majority of genes encoding key enzymes associated with the 3HP bicycle were also significantly upregulated (Fig. 5C and Fig. S4D), strongly indicating the 3HP bicycle was also conducted by strain ZRK33 in the deep-sea environment. Therefore, we speculated that strain ZRK33 performs an autotrophic carbon fixation through the 3HP bicycle, and this pathway could collaborate with phototrophic processes to facilitate the growth of strain ZRK33 in both laboratory and deep-sea environments.

### Wide distribution of genes associated with phototrophy in the deep-sea *Chloroflexi* bacteria.

**FIG 6:**
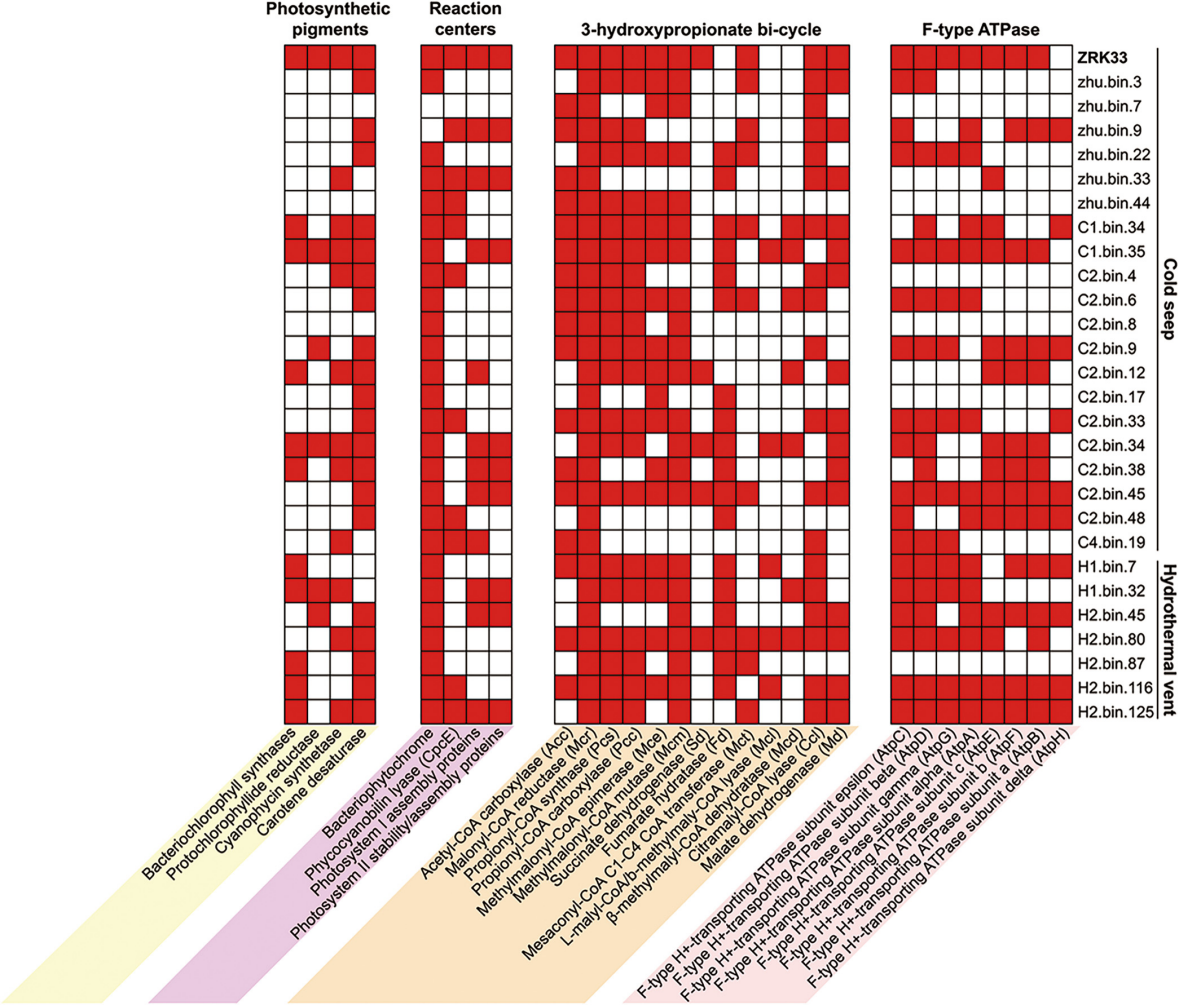
Wide distribution of phototrophic *Chloroflexi* bacteria existing in the deep sea. Shown is an analysis of the distribution of genes encoding key enzymes associated with the phototrophic apparatus and the 3HP bicycle in the genome of strain ZRK33 and MAGs of *Chloroflexi* bacteria derived from a deep-sea cold seep and hydrothermal vent sediments. The names of different MAGs are shown on the right side of the heat map.

To explore the occurrence of the phototrophic lifestyle in the deep-sea *Chloroflexi* bacteria, we further analyzed the distribution of genes encoding key enzymes responsible for phototrophy in 27 metagenome-assembled genomes (MAGs) of *Chloroflexi* bacteria derived from both deep-sea cold seep and hydrothermal vent sediments (Table S4). Through a comprehensive analysis of 27 MAGs as well as the genome of strain ZRK33, we found that diverse genes encoding key proteins closely related to photosynthetic pigments, reaction centers, 3HP bicycle, and F-type ATPase were widely distributed in both cold seep and hydrothermal vent-derived MAGs (Fig. 6). Of note, almost all phototrophy-related genes were present in the genomes of strain ZRK33, C1.bin.34, C1.bin.35, C2.bin.12, C2.bin.34, C2.bin.38, H2.bin.116, and H2.bin.125. Given the high abundance of *Chloroflexi* in both deep-sea cold seeps and hydrothermal vents (Fig. S1), we speculate there are diverse unexplored phototrophic *Chloroflexi* bacteria in the deep-sea environments.

10.1128/mbio.00287-22.9 TABLE S4 Sampling sites for metagenomic analysis and assembly statistics and quality metrics of reconstructed genome bins of *Chloroflexi* used in this study. Download Table S4, DOCX file, 0.02 MB. Copyright © 2022 Zheng et al. 2022 Zheng et al. https://creativecommons.org/licenses/by/4.0/ This content is distributed under the terms of the Creative Commons Attribution 4.0 International license.

## DISCUSSION

Members of the phylum *Chloroflexi* are widely distributed in various environments with high abundance. For example, the number of *Chloroflexi* bacteria was shown to be nearly equivalent to other total bacterial counts in some marine subsurface sediments (32–34). Our OTU sequencing results clearly revealed that *Chloroflexi* bacteria were dominant in both deep-sea cold seeps and hydrothermal vents (see Fig. S1 in the supplemental material). The *Chloroflexi* bacteria catch the attention of many microbiologists due to their photosynthetic lifestyle (35). Recent genomic sequencing projects have expanded the known taxonomic and metabolic diversity of the *Chloroflexi* phylum (9). A monophyletic clade sister to the *Anaerolineae* class is termed “*Ca.* Thermofonsia” (class level) (9). Similar to the classically phototrophic *Chloroflexia* class within the phylum *Chloroflexi*, “*Ca.* Thermofonsia” clade 2 and clade 3 (order level) were also proposed to be novel phototrophs and acquire phototrophy independently via horizontal gene transfer from different ancestral donors within the *Chloroflexia* class (9). However, experimental testing is necessary to confirm inferences about the phototrophic lifestyle of “*Ca.* Thermofonsia.” Therefore, cultivation of the representative of “*Ca.* Thermofonsia” is a priority. In the present study, we developed an innovative enrichment method by keeping a constant rifampin pressure in the basal medium (Fig. 1A) and successfully cultured the first representative (*Phototrophicus methaneseepsis* ZRK33) of “*Ca.* Thermofonsia” clade 2 from a deep-sea cold seep sample (Fig. 1B and 2). Based on comprehensive phylogenetic analyses, we propose “*Ca.* Thermofonsia” as a novel class and strain ZRK33 as a cultured representative. Strain ZRK33 possesses a very high growth rate (4 h for doubling time) compared to other reported deep-sea *Chloroflexi* isolates (6 h to 19 days for doubling time) (28) (Table S1), providing a great advantage for us to promptly perform various assays.

The most attractive trait of *Thermofonsia* clade 2 is the potential phototrophic lifestyle (9). As the first cultured representative of *Thermofonsia* clade 2, strain ZRK33 indeed possesses many phototrophy-associated genes in its genome (Table S2). Moreover, different wavelengths of light (red, infrared, white, green, and blue) could promote the growth rate and biomass of strain ZRK33, especially the red light (Fig. 3). Consistent with this, the expression of most phototrophy-associated genes was significantly upregulated under red light illumination (Fig. 4B), suggesting that strain ZRK33 uses bacteriochlorophyll *a* as the major pigment to absorb the red light (670 to 700 nm) (36), which needs to be verified by *in vivo* absorption spectrum experiments in the future. Additionally, next to red light, the infrared light (940 nm) also evidently promoted the growth of strain ZRK33 (Fig. 3A and B). Similarly, a kind of cyanobacterium collects far-red light (750 nm) through longer-wavelength chlorophyll *f*, which facilitates survival of this bacterium under dark conditions (37). Most importantly, strain ZRK33 also showed a typical phototrophic lifestyle under the deep-sea *in situ* condition (Fig. 4C): the expression of a majority of genes associated with phototrophic apparatus was significantly upregulated when the cells were exposed to the deep-sea potentially illuminous environment. Actually, there is plenty of evidence showing that both long-wavelength (>650 nm) (38, 39) and short-wavelength (<650 nm) (2) light have been detected in deep sea (40). Thus, the necessary conditions for light-associated metabolisms are met in these environments. Accordingly, an obligately photosynthetic bacterial anaerobe (strain GSB1) from a deep-sea hydrothermal vent was capable of conducting photosynthesis by using the long-wavelength (750 nm) geothermal light (3); a nonphototrophic bacterium, Croceicoccus marinus OT19, from a deep-sea hydrothermal vent, could utilize infrared light (940 nm) by the bacteriophytochrome (39). We speculate that strain ZRK33 as well as other deep-sea phototrophic microbes, capable of detecting faint light existing in the deep ocean, could preferentially occupy an optimum habitat and gain evolutionary advantages in the competition for nutrient resources.

In addition, we showed that strain ZRK33 possessed the ability to fix inorganic carbon through the 3HP bicycle (Fig. 5A) under both laboratory (Fig. 5B) and deep-sea conditions (Fig. 5C), strongly suggesting that strain ZRK33 was capable of having an autotrophic life under some conditions. Given strain ZRK33 preferred to grow under a mesophilic condition, we propose the 3HP bicycle exists in both thermophilic and mesophilic *Chloroflexi.* Moreover, the expression pattern of genes associated with the 3HP bicycle (Fig. 5) showed the same trend as those involved in phototrophic metabolism under the illumination condition (Fig. 4), indicating the close relationship between phototrophic and autotrophic pathways. Strain ZRK33 was originally enriched in a basal medium without any organic components (Fig. 1A) but showed a better growth rate in basal medium supplemented with organic substances (e.g., yeast extract or peptone), indicating it is also heterotrophic. Thus, we proposed that strain ZRK33 possessed a presumptive photomixotrophic lifestyle that endowed this bacterium with more flexibility to adapt to and survive under harsh deep-sea conditions.

Notably, a large portion of the genes associated with phototrophic apparatus is widely distributed in the *Chloroflexi* MAGs derived from deep-sea cold seeps and hydrothermal vents (Fig. 6), which strongly suggests that there are diverse phototrophic *Chloroflexi* bacteria in the deep-sea environments. Given the high abundance of *Chloroflexi* bacteria in the deep ocean, we speculate that the contribution of unexplored phototrophic bacteria to the energy cycle has been substantially underestimated.

Taken together, this study expands the variety of cultured *Chloroflexi* as well as the range of possible environments harboring lives that use faint light to drive endergonic biochemical reactions and suggests that phototrophic metabolism is not necessarily limited to solar-illuminated habitats.

## MATERIALS AND METHODS

### Sampling and OTU analysis.

The deep-sea sediment samples were collected by RV *Kexue* from a typical cold seep in the South China Sea (E 119°17'07.322'', N 22°06'58.598'') at a depth of approximately 1,146 m and two hydrothermal vents in the Okinawa Trough (E 126°53'50.247'', N 27°47'11.096''; E 124°22'24.86'', N 25°15'47.438'') in July of 2018. We selected eight sedimentary samples (six cold seep samples, including RPC, ZC1, ZC2, ZC3, ZC4, and ZC5, at depth intervals of 0 to 10, 30 to 50, 90 to 110, 150 to 170, 210 to 230, and 230 to 250 cm, respectively; two hydrothermal vents samples, H1 and H2, at depth intervals of 0 to 20 cm) for OTU sequencing performed by Novogene (Tianjin, China). Briefly, total DNAs from these samples were extracted by a CTAB/SDS method (41) and diluted to 1 ng/μL with sterile water and used for PCR templates. 16S rRNA genes of distinct regions (16S V3/V4) were amplified using the primers (341F, 5′-CCTAYGGGRBGCASCAG; 806R, 5′-GGACTACNNGGGTATCTAAT). The PCR products were purified with a Qiagen gel extraction kit (Qiagen, Germany) for library construction. Sequencing libraries were generated using a TruSeq DNA PCR-free sample preparation kit (Illumina, USA) by following the manufacturer's instructions. The library quality was assessed on the Qubit@ 2.0 fluorometer (Thermo Scientific, USA) and an Agilent Bioanalyzer 2100 system. The library was sequenced on an Illumina NovaSeq platform, and 250-bp paired-end reads were generated. Paired-end reads were merged using FLASH (V1.2.7; http://ccb.jhu.edu/software/FLASH/) (42). Quality filtering on raw tags was performed under specific filtering conditions to obtain the high-quality clean tags (43) according to the QIIME (V1.9.1; http://qiime.org/scripts/split_libraries_fastq.html) quality controlled process. The tags were compared with the reference database (Silva database version 138; https://www.arb-silva.de/) using the UCHIME algorithm (http://www.drive5.com/usearch/manual/uchime_algo.html) (44) to detect chimera sequences (45). Sequence analyses were performed by Uparse software (Uparse v7.0.1001; http://drive5.com/uparse/) (46). Sequences with ≥97% similarity were assigned to the same OTUs. The representative sequence for each OTU was screened for further annotation. The Silva database (http://www.arb-silva.de/) (47) was used based on Mothur algorithm to annotate taxonomic information.

### Enrichment and cultivation of deep-sea *Chloroflexi* bacteria.

To enrich the *Chloroflexi* bacteria, deep-sea sediment samples were cultured at 28°C for 1 month in a basal medium (containing 1.0 g/liter NH_4_Cl, 1.0 g/liter NaHCO_3_, 1.0 g/liter CH_3_COONa, 0.5 g/liter KH_2_PO_4_, 0.2 g/liter MgSO_4_·7H_2_O, 0.7 g/liter cysteine hydrochloride, 500 μL/liter 0.1% [wt/vol] resazurin, pH 7.0) supplemented with 50 μg/mL rifampicin under a 100% N_2_ atmosphere. This basal medium was prepared anaerobically as previously described (48). A 50-μL enrichment culture was spread on the Hungate tubes containing basal medium supplemented with 15 g/liter agar after 10,000 times dilution. These Hungate tubes were anaerobically incubated at 28°C for 7 days. Individual colonies were respectively picked using sterilized bamboo sticks and then cultured in basal medium at 28°C for 5 days under a 100% N_2_ atmosphere. Thereafter, the amplification and sequencing of 16S rRNA genes were performed to identify the species of these cultures. For amplification of 16S rRNA genes, primers 27F (5′-AGAGTTTGATCCTGGCTCAG-3′) and 1492R (5′-GGTTACCTTGTTACGACTT-3′) were used. PCR conditions were the following: predenaturation at 95°C for 10 min; denaturation at 95°C for 15 s, annealing at 55°C for 30 s, extension at 72°C for 30 s, in 30 cycles; and final extension at 72°C for 5 min. These PCR amplification products were sequenced in Tsingke Biotechnology Co., Ltd. (Beijing, China), and the sequences were analyzed by BLAST of the NCBI databases. The *Chloroflexi* strain ZRK33 was selected, isolated, and purified by repeated use of the Hungate roll-tube method for several rounds until it was considered pure. The purity of strain ZRK33 was confirmed by transmission electron microscopy (TEM) and repeated sequencing of the 16S rRNA gene. Finally, strain ZRK33 was preserved at −80°C in basal medium supplemented with 20% (vol/vol) glycerol. Due to the slow growth of strain ZRK33 in the basal medium, additional organic substances were added in basal medium (named rich medium): 1.0 g/liter yeast extract, 1.0 g/liter peptone, 1.0 g/liter CH_3_COONa, 1.0 g/liter NH_4_Cl, 1.0 g/liter NaHCO_3_, 0.5 g/liter KH_2_PO_4_, 0.7 g/liter cysteine hydrochloride, 500 μL/liter of 0.1% (wt/vol) resazurin, 1 liter seawater, pH 7.0.

### TEM observation.

To observe the morphological characteristics of strain ZRK33, the cell suspension of fresh culture was collected at 5,000 × *g* for 10 min and washed with 10 mM phosphate buffer solution (PBS; pH 7.4) and then taken by immersing copper grids coated with a carbon film for 10 min. Thereafter, the copper grids were washed for 10 min in PBS and dried for 20 min at room temperature (49). Ultrathin-section electron microscopic observation was performed as described previously (50). The sample was first preserved in 2.5% (vol/vol) glutaraldehyde for 8 h at 4°C, washed three times with PBS, and then dehydrated in ethanol solutions of 30%, 50%, 70%, 90%, and 100% for 10 min each time. Finally, the sample was embedded in a plastic resin. Ultrathin sections (50 to ∼70 nm) of cells were prepared with an ultramicrotome (Leica EM UC7; Germany), stained with uranyl acetate and lead citrate. All of these samples were examined using TEM (HT7700; Hitachi, Japan) with a JEOL JEM 12000 EX (equipped with a field emission gun) at 100 kV.

### Genome sequencing and analysis.

Genomic DNA of strain ZRK33 was extracted from 1.5 liters of cells that were cultured for 7 days at 28°C. The DNA library was prepared using a ligation sequencing kit (SQK-LSK109; UK) and sequenced using a FLO-MIN106 vR9.4 flow cell for 48 h on MinKNOWN software v1.4.2 (Oxford Nanopore Technologies, UK). Base-calling was performed using Albacore software v2.1.10 (Oxford Nanopore Technologies, UK). Nanopore reads were processed using a protocol toolkit for quality control and downstream analysis (51). Filtered reads were assembled using Canu version 1.8 (52) with the default parameters for Nanopore data. Finally, the genome was assembled into a single contig and was manually circularized by deleting an overlapping end.

The genome relatedness values were calculated by multiple approaches: average nucleotide identity based on the MUMMER ultrarapid aligning tool (ANIm), ANI based on the BLASTN algorithm (ANIb), the tetranucleotide signatures (Tetra), and *in silico* DNA-DNA similarity. ANIm, ANIb, and Tetra frequencies were calculated using JSpecies WS (http://jspecies.ribohost.com/jspeciesws/) (53). The recommended species criterion cutoffs were used: 95% for ANIb and ANIm and 0.99 for the Tetra signature. The *in silico* DNA-DNA similarity values were calculated by the Genome-to-Genome Distance Calculator (GGDC) (http://ggdc.dsmz.de/) (54). The *is*DDH results were based on the recommended formula 2, which is independent of genome size. The prediction of photosynthesis-associated genes in the genome of strain ZRK33 and MAGs of deep-sea *Chloroflexi* was performed using Galaxy version 2.6.0 (https://galaxy.pasteur.fr/) (55) with the NCBI BLASTP method.

### Phylogenetic analysis.

The maximum likelihood phylogenetic tree based on the genomes was constructed from a concatenated alignment of 37 protein-coding genes (56) that was extracted from each genome by Phylosift (v1.0.1) (57), all of which were in a single copy and universally distributed in both archaea and bacteria. The genomes used to construct the genome tree included both draft and finished genomes from the NCBI databases (https://www.ncbi.nlm.nih.gov/). The maximum likelihood phylogenetic tree of the 16S rRNA gene was constructed with the full-length 16S rRNA sequences, which were all obtained from the NCBI database. All the sequences were aligned by MAFFT version 7 (58) and manually corrected. The phylogenetic tree was constructed by using the W-IQ-TREE web server (http://iqtree.cibiv.univie.ac.at) (59) with the LG+F+I+G4 model and was edited by the online tool Interactive Tree of Life (iTOL v5) (60).

### Phenotypic characteristics analyses.

For phenotypic characteristics analyses, the temperature, pH, and NaCl concentration ranges for the growth of strain ZRK33 were determined in rich medium. Growth was tested at different temperatures (4, 16, 28, 30, 32, 37, 45, 60, 70, and 80°C) for 15 days. The pH range for growth was tested on rich medium from pH 2.0 to pH 12.0 with increments of 0.5 pH units. Salt tolerance was tested on basal medium (replacing seawater with distilled water) supplemented with 0 to 10% (wt/vol) NaCl (0.5% intervals) at 28°C for 15 days. Aerobic growth was tested on a 2216E agar plate (5 g/liter peptone, 1 g/liter yeast extract, and 15 g/liter agar) for 15 days at 28°C. Substrate utilization of strain ZRK33 was tested in the medium consisting of (liter^−1^) 5 g NaCl, 1.0 g NH_4_Cl, 0.5 g KH_2_PO_4_, 0.2 g MgSO_4_, 0.02 g yeast extract. A single substrate (including arabinose, fructose, glucose, galactose, mannose, ribose, xylose, fumarate, pyruvate, and peptone) was added to this medium to a final concentration at 20 mM. The medium (containing only 0.02 g/liter yeast extract) without any added substrates was used as the control group. Finally, these cultures were incubated at 28°C for 15 days and then examined by spectrophotometry at 600 nm. For each substrate, the corresponding assay was repeated three times. For cellular respiratory quinones, strain ZRK33 was inoculated in 2-liter Hungate bottles containing 1.5 liters of rich medium for 7 days at 28°C. The cellular fatty acids were extracted and identified by using gas chromatography (model 7890A; Agilent, USA) according to the protocol of the Sherlock Microbial Identification System.

### Growth assay and morphological observation of *P. methaneseepsis* ZRK33 under darkness and different lights.

Growth assays were performed at an atmospheric pressure. Briefly, 15 mL fresh strain ZRK33 culture was inoculated in 2-liter Hungate bottles containing 1.5 liters of rich medium under darkness and illumination with different wavelengths of light (white, green, blue, infrared, and red). Three replicates were conducted for each assay. The Hungate bottles were anaerobically incubated at 28°C for 12 days. Bacterial growth status was monitored by measuring the optical density at 600 nm (OD_600_) each day until cell growth reached the stationary phase. For the morphological observation of strain ZRK33, 5 μL cultured cells at the late log phase under different cultivation conditions were taken and then observed and recorded by an inverted microscope (NIKON TS100; Tokyo, Japan) equipped with a digital camera. In parallel, the cells’ length and number under each condition were calculated.

### Transcriptomic analysis of *P. methaneseepsis* ZRK33 cultured under darkness and light illumination.

For transcriptomic analysis, cells of strain ZRK33 cultured in 1.5 liters of basal medium under darkness and illumination with different wavelengths of light (white, green, blue, infrared, and red) for 7 days was collected at 8,000 × *g* for 20 min. Afterwards, respective cells were collected, and the transcriptomic analysis was conducted by Novogene (Tianjin, China). Briefly, a total amount of 3 μg RNA per sample was used as input material for the RNA sample preparations. Sequencing libraries were generated using NEBNext Ultra directional RNA library prep kit for Illumina (NEB, USA) by following the manufacturer’s recommendations, and index codes were added to attribute sequences to each sample. The clustering of the index-coded samples was performed on a cBot Cluster Generation System using TruSeq PE Cluster kit v3-cBot-HS (Illumia) according to the manufacturer’s instructions. After cluster generation, the library preparations were sequenced on an Illumina HiSeq platform and paired-end reads were generated. Raw data (raw reads) of fastq format were first processed through in-house Perl scripts. In this step, clean data (clean reads) were obtained by removing reads containing adapter, reads containing poly-N, and low-quality reads from raw data. Both building index of reference genome and aligning clean reads to reference genome used Bowtie2-2.3.4.3 (settings: -D 15 -R 2 -N 0 -L 22 -i S, 1, 1.15) (61). HTSeq v0.6.1 (default parameters) was used to count the read numbers mapped to each gene. The fragments per kilobase per million of each gene was calculated based on the length of the gene and read counts mapped to this gene (62). Differential expression analysis of two conditions/groups was performed using the DESeq R package (1.18.0) and edgeR v3.24.3 (settings: |log_2_(fold change)| ≥ 1, padj ≤ 0.05) (63). The resulting *P* values were adjusted using the Benjamini and Hochberg’s approach for controlling the false discovery rate. Genes with an adjusted *P* value of <0.05 were assigned as differentially expressed. Corrected *P* value of 0.005 and log_2_(fold change) of 1 were set as the threshold for significant differential expression. The detailed protocols of library preparation, clustering, and sequencing and data analyses are described in Text S1.

### Transcriptomic analysis of *P. methaneseepsis* ZRK33 cultured in deep-sea cold seep.

To explore the potential phototrophic lifestyle of strain ZRK33 conducted in the deep-sea cold seep, *in situ* cultivation was performed. Briefly, strain ZRK33 was first cultured in rich medium for 7 days, and then the culture was divided into two parts: one part was equally transferred to three nontransparent anaerobic bags (which prevented any light exposure; Hede, China) with 200 mL rich medium each and set as the control groups; the other part was divided into three transparent anaerobic bags (which allowed light; Hede, China) with 200 mL rich medium each and set as experimental groups. All the anaerobic bags were placed simultaneously in the deep-sea cold seep where strain ZRK33 was isolated for 10 days in June 2021 during the cruise of the *Kexue*. After 10 days of *in situ* cultivation, these bags were collected. Cells of strain ZRK33 in corresponding bags were immediately collected and kept in a −80°C freezer for future analysis. Before transcriptomic analysis, the cells in each bag were checked by 16S rRNA gene sequencing to confirm the purity. The detailed protocol for transcriptomic sequencing was performed as described above.

### Metagenomic sequencing, assembly, binning, and annotation.

To explore the existence of photosynthesis-associated genes in the metagenomes of *Chloroflexi* in deep-sea environments, four cold seep sediment samples (zhu, C1, C2, and C4) and two hydrothermal vents sediment samples (H1 and H2) were selected for metagenomic analysis in BGI (Qingdao, China). Briefly, total DNAs from these samples (20 g each) were extracted using the Qiagen DNeasy PowerSoil Pro kit (Qiagen, Germany), and the integrity of the DNA was evaluated by gel electrophoresis. Next, 0.5 μg DNA of each sample was used for library construction. The library was prepared with an amplification step for each sample. DNAs were cleaved into 50- to 800-bp fragments by the Covaris E220 ultrasonicator (Covaris, Brighton, UK), and some fragments between 150 and 250 bp were selected using AMPure XP beads (Agencourt, USA) and repaired using T4 DNA polymerase (Enzymatics, USA). All next-generation sequencing (NGS) was performed on the BGISEQ-500 platform (BGI) and generated 100-bp paired-end raw reads. Quality control was performed by SOAPnuke (v1.5.6) (settings: -l 20 -q 0.2 -n 0.05 -Q 2 -d -c 0–5 0–7 1) (64), and the clean data were assembled using MEGAHIT (v1.1.3) (settings: –min-count 2 –k-min 33 –k-max 83 –k-step 10) (65). Assemblies of these samples were automatically binned using Maxbin2 (66), metaBAT2 (67), and Concoct (68). MetaWRAP (69) was used to purify and organize data to generate the final bins. Finally, the completeness and contamination of MAGs were assessed by checkM (v1.0.18) (70). These obtained MAGs were subsequently annotated by searching these predicted genes against KEGG (release 87.0), NR (20180814), Swiss-Prot (release-2017_07), and COG (update-2018_08) databases. Additionally, we utilized a custom hmmer as well as the Pfam and TIGRFAM databases to search for genes associated with photosynthesis using hmmsearch (E value cutoff of 1e−20) (71).

### Data availability.

The raw amplicon sequencing data have been deposited in the NCBI Short Read Archive (accession numbers PRJNA675395 and PRJNA688815). The BioProject accession number of MAGs of *Chloroflexi* bacteria used in this study is PRJNA667788. The full-length 16S rRNA gene sequence of *P. methaneseepsis* ZRK33 has been deposited at GenBank under the accession number MN817941. The complete genome sequence of *P. methaneseepsis* ZRK33 has been deposited at GenBank under the accession number CP051151. The raw sequencing reads from the transcriptomics analysis have been deposited in the NCBI Short Read Archive (accession numbers PRJNA685946 and PRJNA758590).

10.1128/mbio.00287-22.10 TABLE S5 Primers used for qRT-PCR. Download Table S5, DOCX file, 0.01 MB. Copyright © 2022 Zheng et al. 2022 Zheng et al. https://creativecommons.org/licenses/by/4.0/ This content is distributed under the terms of the Creative Commons Attribution 4.0 International license.
