# Erratum for Zheng et al., “Characterization of the First Cultured Representative of ‘*Candidatus* Thermofonsia’ Clade 2 within *Chloroflexi* Reveals Its Phototrophic Lifestyle”

**DOI:** 10.1128/mbio.00657-22

**Published:** 2022-03-21

**Authors:** Rikuan Zheng, Ruining Cai, Chong Wang, Rui Liu, Chaomin Sun

**Affiliations:** a CAS and Shandong Province Key Laboratory of Experimental Marine Biology & Center of Deep Sea Research, Institute of Oceanology, Chinese Academy of Sciences, Qingdao, China; b Laboratory for Marine Biology and Biotechnology, Qingdao National Laboratory for Marine Science and Technology, Qingdao, China; c College of Earth Science, University of Chinese Academy of Sciences, Beijing, China; d Center of Ocean Mega-Science, Chinese Academy of Sciences, Qingdao, China

## ERRATUM

Volume 13, no. 2, e00287-22, 2022, https://doi.org/10.1128/mbio.00287-22. Page 7: In Fig. 4A, the label H^+^+NADPH should be H^+^+NADP^+^. The corrected figure appears here. Page 14: In the “Data availability” paragraph, accession number CP051151 should be CP062983.

**FIG 4:**
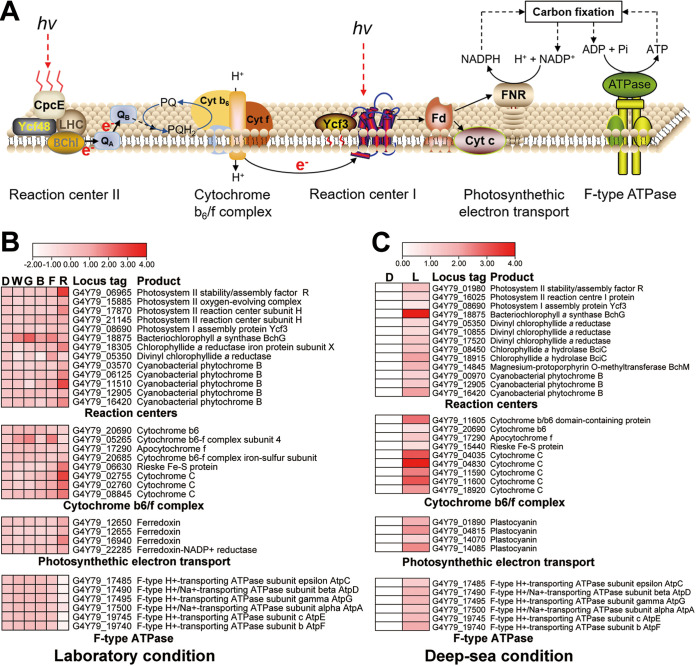
Transcriptomic analysis of the phototrophic lifestyle conducted by *P. methaneseepsis* ZRK33 under both laboratory and deep-sea conditions.

