# Supplementary material for: Characterization of the First Cultured Representative of “Candidatus Thermofonsia” Clade 2 within Chloroflexi Reveals Its Phototrophic Lifestyle
Source: mBio. 2022 Mar 1;13(2):e00287-22. doi: 10.1128/mbio.00287-22 (PMC8941918; doi:10.1128/mbio.00287-22)
Supplement: FIG S1 [file mbio.00287-22-sf001.docx]

**
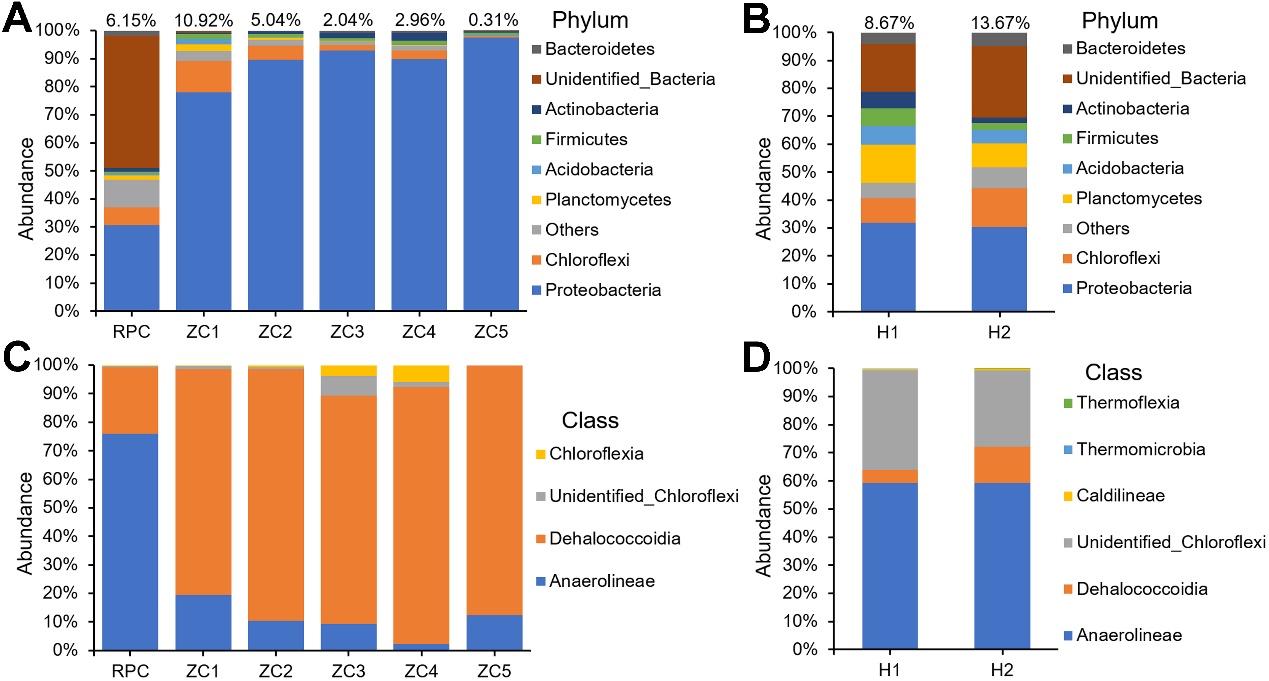
**

**FIG S1.** Detection of the abundance of the phylum *Chloroflexi* derived from deep-sea cold seeps and hydrothermal vents sediments. The community structure of six sampling sites in the cold seep sediments and two sampling sites in the hydrothermal vents sediments as revealed by 16S rRNA gene amplicon profiling. The relative abundances of OTUs representing different bacteria are shown at the phylum level (A and B) and class level (C and D). Panels A and C represent samples from the cold seep; panels B and D represent samples from the hydrothermal vents.
