# Supplementary material for: Characterization of the First Cultured Representative of “Candidatus Thermofonsia” Clade 2 within Chloroflexi Reveals Its Phototrophic Lifestyle
Source: mBio. 2022 Mar 1;13(2):e00287-22. doi: 10.1128/mbio.00287-22 (PMC8941918; doi:10.1128/mbio.00287-22)
Supplement: FIG S2 [file mbio.00287-22-sf002.docx]

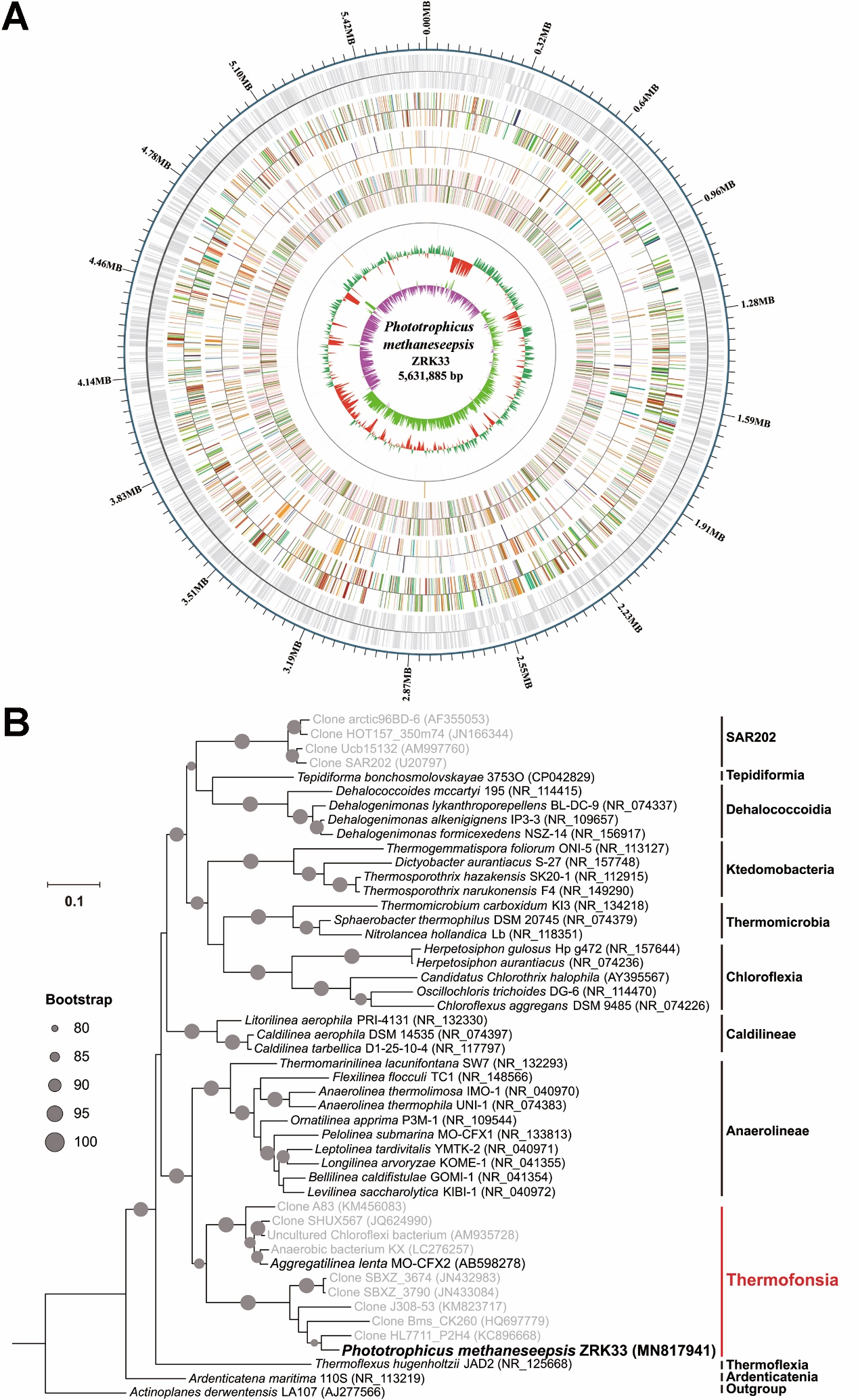


**FIG S2.** Genomic map and phylogenetic analysis of strain ZRK33. (A) Circular diagram of the genome of strain ZRK33. Rings indicate, from outside to the center: a genome-wide marker with a scale of 320 kb; forward strand genes, colored by COG category; reverse strand genes, colored by COG category; gene function annotation (COG, KEGG, GO, NR, CAZy, TCDB); RNA genes (tRNAs blue, rRNAs purple); GC content; GC skew. (B) Maximum likelihood phylogenetic tree of 16S rRNA gene sequences from strain ZRK33 and other *Chloroflexi* bacteria. The tree is inferred and reconstructed under the maximum likelihood criterion and bootstrap values (%) > 80 are indicated at the base of each node with grey dots (expressed as percentages of 1,000 replications). Names indicated with grey color in quotation represent taxa that are not yet validly published. All sequences are labeled with their NCBI accession numbers. The 16S rRNA gene sequence of *Actinoplanes derwentensis* LA107 is used as an outgroup. Bar, 0.1 substitutions per nucleotide position.
