# Supplementary material for: Characterization of the First Cultured Representative of “Candidatus Thermofonsia” Clade 2 within Chloroflexi Reveals Its Phototrophic Lifestyle
Source: mBio. 2022 Mar 1;13(2):e00287-22. doi: 10.1128/mbio.00287-22 (PMC8941918; doi:10.1128/mbio.00287-22)
Supplement: TABLE S1 [file mbio.00287-22-st001.docx]

**Supplementary Table S1.** Characteristics of strain ZRK33 and the other isolated *Chloroflexi* members. Strains: 1, strain ZRK33; 2, *Aggregatilinea lenta* MO-CFX2^T^ (1); 3, *Pelolinea submarina* MO-CFX1^T^ (2); 4, *Anaerolinea thermophila* UNI-1^T^ (3); 5, *Anaerolinea thermolimosa* IMO-1^T^ (4); 6, *Ornatilinea apprima* P3M-1^T^ (5). +, Positive; -, negative; NA, no data available.

| **Characteristic** | **1** | **2** | **3** | **4** | **5** | **6** |
| --- | --- | --- | --- | --- | --- | --- |
| Cell morphology  Cell diameter (µm)  Optimum temperature for growth (°C)  Optimum pH for growth  NaCl concentration for growth (%)  Draft (or complete)  genome size (Mbp)  ANIb (%)  ANIm (%)  Tetra  GGDC (%)  DNA G+C content (%)  Major cellular  fatty acids  Doubling time  Substrates for growth:  Arabinose  Fructose  Glucose  Galactose  Mannose  Ribose  Xylose  Fumarate  Pyruvate  Peptone  Isolation source | Filaments  0.3-0.5  28  7.0  0-5  5.6  100  100  1  100  52.76  C_16:0_, C_15:0_2-OH,  C_17:1_*ω*6c,  C_18:1_*ω*7c  4 h  +  +  +  +  +  +  -  +  +  +  Deep-sea  cold seep  sediments | Filaments  0.5-0.6  30  6.5-7.0  0-3  6.2  64.81  85.21  0.48145  23.30  63.2  C_16:0_, C_18:0_, C_18:1_*ω*9c  19 days  -  -  -  -  -  -  -  +  +  -  Marine  subsurface  sediment | Filaments  0.13-0.15  25-30  7.0  0-5  3.5  63.06  82.63  0.67572  24.20  50.6  C_18:1_*ω*9, C_16:1_*ω*7, i-C_17:0_3-OH, C_16:0_  1.5 days  +  +  +  +  -  +  +  -  -  -  Marine subsurface  sediment | Filaments  0.2-0.3  55  7.0  0-5  3.5  63.42  83.42  0.64677  20.40  53.8  C_16:0_,  C_15:0_, C_14:0_  3 days  +  +  +  +  +  +  +  -  +  NA  Thermophilic  anaerobic  sludge | Filaments  0.3-0.4  50  7.0  0-2.5  4.2  63.41  83.15  0.65234  21.60  53.7  ai-C_17:0_, i-C_15:0_, C_16:0_  2 days  +  +  +  +  +  +  +  -  +  +  Thermophilic  anaerobic  sludge | Filaments  0.3-0.7  42-45  7.5-8.0  0-2  4.4  63.29  83.23  0.65126  23.80  55.7  i-C_15:0_, ai-C_15:0_, C_14:0_  6 h  -  -  +  -  NA  NA  +  -  NA  -  Deep terrestrial hot aquifer |

**References related to this table**

1. Nakahara N, Nobu MK, Takaki Y, Miyazaki M, Tasumi E, Sakai S, Ogawara M, Yoshida N, Tamaki H, Yamanaka Y, Katayama A, Yamaguchi T, Takai K, Imachi H. 2019. *Aggregatilinea lenta* gen. nov., sp. nov., a slow-growing, facultatively anaerobic bacterium isolated from subseafloor sediment, and proposal of the new order *Aggregatilineales* ord. nov. within the class *Anaerolineae* of the phylum *Chloroflexi*. Int J Syst Evol Microbiol 69:1185-1194.

2. Imachi H, Sakai S, Lipp JS, Miyazaki M, Saito Y, Yamanaka Y, Hinrichs KU, Inagaki F, Takai K. 2014. *Pelolinea submarina* gen. nov., sp. nov., an anaerobic, filamentous bacterium of the phylum *Chloroflexi* isolated from subseafloor sediment. Int J Syst Evol Microbiol 64:812-818.

3. Sekiguchi Y, Yamada T, Hanada S, Ohashi A, Harada H, Kamagata Y. 2003. *Anaerolinea thermophila* gen. nov., sp. nov. and *Caldilinea aerophila* gen. nov., sp. nov., novel filamentous thermophiles that represent a previously uncultured lineage of the domain Bacteria at the subphylum level. Int J Syst Evol Microbiol 53:1843-51.

4. Yamada T, Sekiguchi Y, Hanada S, Imachi H, Ohashi A, Harada H, Kamagata Y. 2006. *Anaerolinea thermolimosa* sp. nov., *Levilinea saccharolytica* gen. nov., sp. nov. and *Leptolinea tardivitalis* gen. nov., sp. nov., novel filamentous anaerobes, and description of the new classes *Anaerolineae* classis nov. and *Caldilineae* classis nov. in the bacterial phylum *Chloroflexi*. Int J Syst Evol Microbiol 56:1331-1340.

5. Podosokorskaya OA, Bonch-Osmolovskaya EA, Novikov AA, Kolganova TV, Kublanov IV. 2013. *Ornatilinea apprima* gen. nov., sp. nov., a cellulolytic representative of the class *Anaerolineae*. Int J Syst Evol Microbiol 63:86-92.
