# Supplementary material for: Characterization of the First Cultured Representative of “Candidatus Thermofonsia” Clade 2 within Chloroflexi Reveals Its Phototrophic Lifestyle
Source: mBio. 2022 Mar 1;13(2):e00287-22. doi: 10.1128/mbio.00287-22 (PMC8941918; doi:10.1128/mbio.00287-22)
Supplement: TEXT S1 [file mbio.00287-22-s0001.docx]

**SUPPLEMENTAL MATERIAL**

**A detailed procedure for transcriptomic sequencing analysis of *P. methaneseepsis* ZRK33 cultured under different conditions.**

**(1) Library preparation for strand-specific transcriptome sequencing.** A total amount of 3 μg RNA per sample was used as input material for the RNA sample preparations. Sequencing libraries were generated using NEBNext^®^ Ultra™ Directional RNA Library Prep Kit for Illumina^®^ (NEB, USA) following manufacturer’s recommendations and index codes were added to attribute sequences to each sample. rRNA is removed using a specialized kit that leaves the mRNA. Fragmentation was carried out using divalent cations under elevated temperature in NEBNext First Strand Synthesis Reaction Buffer (5×). First strand cDNA was synthesized using random hexamer primer and M-MuLV Reverse Transcriptase (RNaseH^-^). Second strand cDNA synthesis was subsequently performed using DNA Polymerase I and RNase H. In the reaction buffer, dNTPs with dTTP were replaced by dUTP. Remaining overhangs were converted into blunt ends via exonuclease/polymerase activities. After adenylation of 3’ ends of DNA fragments, NEBNext Adaptor with hairpin loop structure was ligated to prepare for hybridization. In order to select cDNA fragments of preferentially 150~200 bp in length, the library fragments were purified with AMPure XP system (Beckman Coulter, Beverly, USA). Then 3 μL USER Enzyme (NEB，USA) was used with size-selected, adaptor-ligated cDNA at 37 °C for 15 min followed by 5 min at 95 °C before PCR. Then PCR was performed with Phusion High-Fidelity DNA polymerase, Universal PCR primers and Index (X) Primer. At last, products were purified (AMPure XP system) and library quality was assessed on the Agilent Bioanalyzer 2100 system.

**(2) Clustering and sequencing.** The clustering of the index-coded samples was performed on a cBot Cluster Generation System using TruSeq PE Cluster Kit v3-cBot-HS (Illumia) according to the manufacturer’s instructions. After cluster generation, the library preparations were sequenced on an Illumina Hiseq platform and paired-end reads were generated.

**(3) Data analysis.** Raw data (raw reads) of fastq format were firstly processed through in-house perl scripts. In this step, clean data (clean reads) were obtained by removing reads containing adapter, reads containing ploy-N and low quality reads from raw data. At the same time, Q20, Q30 and GC content the clean data were calculated. All the downstream analyses were based on the clean data with high quality. Reference genome and gene model annotation files were downloaded from genome website directly. Both building index of reference genome and aligning clean reads to reference genome were used Bowtie2-2.2.3 (setting: -D 15 -R 2 -N 0 -L 22 -i S,1,1.15) (1). HTSeq v0.6.1 (default parameters) was used to count the reads numbers mapped to each gene. And then FPKM of each gene was calculated based on the length of the gene and reads count mapped to this gene. FPKM, expected number of Fragments Per Kilobase of transcript sequence per Millions base pairs sequenced, considers the effect of sequencing depth and gene length for the reads count at the same time, and is currently the most commonly used method for estimating gene expression levels (2).

**(4) Differential expression analysis.** Differential expression analysis of two conditions/groups (two biological replicates per condition) was performed using the DESeq R package (1.18.0) and edgeR v3.24.3 (|log_2_(FoldChange)| >= 1 & padj <= 0.05) (3). DESeq provide statistical routines for determining differential expression in digital gene expression data using a model based on the negative binomial distribution. The resulting *P*-values were adjusted using the Benjamini and Hochberg’s approach for controlling the false discovery rate. Genes with an adjusted *P*-value < 0.05 found by DESeq were assigned as differentially expressed. (For DEGSeq without biological replicates) Prior to differential gene expression analysis, for each sequenced library, the read counts were adjusted by edgeR program package through one scaling normalized factor. Differential expression analysis of two conditions was performed using the DEGSeq R package (1.20.0) (4). The *P* values were adjusted using the Benjamini & Hochberg method. Corrected *P*-value of 0.005 and log_2_ (Fold change) of 1 were set as the threshold for significantly differential expression.

**(5) GO and KEGG enrichment analysis of differentially expressed genes.** Gene Ontology (GO) enrichment analysis of differentially expressed genes was implemented by the GOseq R package, in which gene length bias was corrected (5). GO terms with corrected *P* value less than 0.05 were considered significantly enriched by differential expressed genes. KEGG is a database resource for understanding high-level functions and utilities of the biological system, such as the cell, the organism and the ecosystem, from molecular-level information, especially large-scale molecular datasets generated by genome sequencing and other high-throughput experimental technologies (http://www.genome.jp/kegg/) (6). We used KOBAS software to test the statistical enrichment of differential expression genes in KEGG pathways.

**Real-Time Quantitative Reverse Transcription PCR (**qRT-PCR**).**

For qRT-PCR, cells of strain ZRK33 cultured in 1.5 L basal medium under darkness and illumination for 7 days in the laboratory condition, and cells cultured in deep-sea cold seep for 10 days were collected, respectively. Total RNAs from each sample were extracted using the Trizol reagent (Solarbio, China) and the RNA concentration was measured using Qubit® RNA Assay Kit in Qubit® 2.0 Flurometer (Life Technologies, CA, USA). Then RNAs from corresponding sample were reverse transcribed into cDNA and the transcriptional levels of different genes were determined by qRT-PCR using SybrGreen Premix Low rox (MDbio, China) and the QuantStudioTM 6 Flex (Thermo Fisher Scientific, USA). The PCR condition was set as following: initial denaturation at 95 °C for 3 min, followed by 40 cycles of denaturation at 95 °C for 10 s, annealing at 60 °C for 30 s, and extension at 72 °C for 30 s. 16S rRNA gene was used as an internal reference and the gene expression was calculated using the 2^-ΔΔCt^ method, with each transcript signal normalized to that of 16S rRNA. Transcript signals for each treatment were compared to those of control group. Speciﬁc primers for genes associated with phototrophic apparatus and 3HP bicycle of strain ZRK33 and 16S rRNA gene were designed using Primer 5.0 as shown in Supplementary Table S5.

**References related to the supplementary method**

1. Langmead B, Salzberg SL. 2012. Fast gapped-read alignment with Bowtie 2. Nat Methods 9:357-U54.

2. Trapnell C, Pachter L, Salzberg SL. 2009. TopHat: discovering splice junctions with RNA-Seq. Bioinformatics 25:1105-1111.

3. Anders S, Huber W. 2010. Differential expression analysis for sequence count data. Genome Biol 11.

4. Wang LK, Feng ZX, Wang X, Wang XW, Zhang XG. 2010. DEGseq: an R package for identifying differentially expressed genes from RNA-seq data. Bioinformatics 26:136-138.

5. Young MD, Wakefield MJ, Smyth GK, Oshlack A. 2010. Gene ontology analysis for RNA-seq: accounting for selection bias. Genome Biol 11.

6. Kanehisa M, Araki M, Goto S, Hattori M, Hirakawa M, Itoh M, Katayama T, Kawashima S, Okuda S, Tokimatsu T, Yamanishi Y. 2008. KEGG for linking genomes to life and the environment. Nucleic Acids Res 36:D480-D484.

**SUPPLEMENTARY RESULTS**

**Description of *Phototrophicaceae* fam. nov.** *Phototrophicaceae* (Pho.to'tro.phi.ca.ce.ae. L. fem. n. *Phototrophicus* type genus of the family; suff. -aceae, ending to denote a family; N.L. fem. pl. n. *Phototrophicaceae* the *Phototrophicus* family).

The description is the same as that for the genus *Phototrophicus*. The type genus is *Phototrophicus*.

**Description of *Phototrophicales* ord. nov.** *Phototrophicales* (Pho.to'tro.phi.ca.les. L. fem. n. *Phototrophicus* type genus of the order; suff. -ales ending to denote an order; N.L. fem. pl. n. *Phototrophicales* order of the genus *Phototrophicus*).

The description is the same as that for the genus *Phototrophicus*. The type genus is *Phototrophicus*.
