# Supplementary material for: Characterization of the First Cultured Representative of “Candidatus Thermofonsia” Clade 2 within Chloroflexi Reveals Its Phototrophic Lifestyle
Source: mBio. 2022 Mar 1;13(2):e00287-22. doi: 10.1128/mbio.00287-22 (PMC8941918; doi:10.1128/mbio.00287-22)
Supplement: TABLE S2 [file mbio.00287-22-st002.docx]

**Supplementary Table S2.** Phototrophy-associated genes in the genome of strain ZRK33.

| **Gene id** | **Protein** | **Gene name** |
| --- | --- | --- |
| **Photosystem** |  |  |
| G4Y79_01705 | Photosystem II stability/assembly factor R |  |
| G4Y79_01765 | Photosystem II stability/assembly factor R |  |
| G4Y79_01980 | Photosystem II reaction center protein |  |
| G4Y79_06965 | Photosystem II stability/assembly factor R |  |
| G4Y79_12095 | Photosystem II reaction center protein |  |
| G4Y79_15885 | Photosystem II oxygen-evolving complex |  |
| G4Y79_16025 | Photosystem II reaction center I protein |  |
| G4Y79_17870 | Photosystem II reaction center subunit H |  |
| G4Y79_21145 | Photosystem II reaction center subunit H |  |
| G4Y79_17860 | Light harvesting complexⅡ | *LHCⅡ* |
| G4Y79_08690 | Photosystem I assembly protein | *Ycf3* |
| G4Y79_13015 | Bacteriochlorophyll c/d synthase | *BchK* |
| G4Y79_22280 | Geranylgeranyl-bacteriochlorophyllide a reductase | *BchP* |
| G4Y79_18875 | Chlorophyll/bacteriochlorophyll a/b synthase | *BchG* |
| G4Y79_02950 | Bacteriochlorophyllide a dehydrogenase | *BhcC* |
| G4Y79_18305 | 3,8-divinyl chlorophyllide a/chlorophyllide a reductase subunit X | *BchX* |
| G4Y79_05350 | Divinyl chlorophyllide a reductase; 3,8-Divinyl chlorophyllide a | *DVR* |
| G4Y79_10855 | Divinyl chlorophyllide a; 3,8-Divinyl chlorophyllide a | *DVR* |
| G4Y79_17815 | Divinyl chlorophyllide a; 3,8-Divinyl chlorophyllide a | *DVR* |
| G4Y79_17520 | Divinyl chlorophyllide a; 3,8-Divinyl chlorophyllide a | *DVR* |
| G4Y79_19020 | Bacteriochlorophyllide d C-8(2)-methyltransferase | *BchQ* |
| G4Y79_08450 | Chlorophyllide a hydrolase | *BciC* |
| G4Y79_18915 | Chlorophyllide a hydrolase | *BciC* |
| G4Y79_14845 | Magnesium-protoporphyrin O-methyltransferase | *BchM* |
| G4Y79_02280 | Magnesium-protoporphyrin O-methyltransferase | *BchM* |
| G4Y79_00460 | Magnesium-protoporphyrin O-methyltransferase | *BchM* |
| G4Y79_23670 | Magnesium-protoporphyrin O-methyltransferase | *BchM* |
| G4Y79_17320 | Mg-protoporphyrin IX monomethyl ester oxidative cyclase | *ChlE* |
| G4Y79_21745 | Mg-protoporphyrin IX monomethyl ester oxidative cyclase | *ChlE* |
| G4Y79_00970 | Cyanobacterial phytochrome B |  |
| G4Y79_03570 | Cyanobacterial phytochrome B |  |
| G4Y79_06125 | Cyanobacterial phytochrome B |  |
| G4Y79_11510 | Cyanobacterial phytochrome B |  |
| G4Y79_12905 | Cyanobacterial phytochrome B |  |
| G4Y79_16420 | Cyanobacterial phytochrome B |  |
| G4Y79_16135 | Phycocyanobilin lyase subunit alpha | *CpcE* |
| **Cytochrome *b6/f* complex** | |  |
| G4Y79_20690 | Cytochrome b6 |  |
| G4Y79_05265 | Cytochrome b6-f complex subunit 4 |  |
| G4Y79_17290 | Apocytochrome f |  |
| G4Y79_01670 | Cytochrome b6-f complex iron-sulfur subunit |  |
| G4Y79_20685 | Cytochrome b6-f complex iron-sulfur subunit |  |
| G4Y79_13765 | Cytochrome b6-f complex subunit 5 |  |
| G4Y79_06630 | Rieske Fe-S protein |  |
| G4Y79_06920 | Rieske Fe-S protein |  |
| G4Y79_15440 | Rieske Fe-S protein |  |
| **Photosynthetic electron transport system** | |  |
| G4Y79_01890 | Plastocyanin |  |
| G4Y79_04815 | Plastocyanin |  |
| G4Y79_14070 | Plastocyanin |  |
| G4Y79_14085 | Plastocyanin |  |
| G4Y79_18880 | Plastocyanin |  |
| G4Y79_03375 | Ferredoxin |  |
| G4Y79_06240 | Ferredoxin |  |
| G4Y79_06955 | Ferredoxin |  |
| G4Y79_12650 | Ferredoxin |  |
| G4Y79_12655 | Ferredoxin |  |
| G4Y79_14935 | Ferredoxin |  |
| G4Y79_16940 | Ferredoxin |  |
| G4Y79_22455 | Ferredoxin |  |
| G4Y79_22285 | Ferredoxin--NADP+ reductase |  |
| **F-ATPase** |  |  |
| G4Y79_17485 | F-type H+-transporting ATPase subunit epsilon | *AtpC* |
| G4Y79_17490 | F-type H+-transporting ATPase subunit beta | *AtpD* |
| G4Y79_17495 | F-type H+-transporting ATPase subunit gamma | *AtpG* |
| G4Y79_17500 | F-type H+-transporting ATPase subunit alpha | *AtpA* |
| G4Y79_19745 | F-type H+-transporting ATPase subunit c | *AtpE* |
| G4Y79_19740 | F-type H+-transporting ATPase subunit b | *AtpF* |
| **3-Hydroxypropionate cycle (3HP bicycle)** | |  |
| G4Y79_19465 | Acetyl-CoA carboxylase | *Acc* |
| G4Y79_21820 | Acetyl-CoA carboxylase | *Acc* |
| G4Y79_19380 | Malonyl-CoA reductase | *Mcr* |
| G4Y79_19325 | Malonyl-CoA reductase | *Mcr* |
| G4Y79_07100 | Malonyl-CoA reductase | *Mcr* |
| G4Y79_12485 | Malonyl-CoA reductase | *Mcr* |
| G4Y79_21855 | Malonyl-CoA reductase | *Mcr* |
| G4Y79_12315 | Malonyl-CoA reductase | *Mcr* |
| G4Y79_08855 | Propionyl-CoA synthase | *Pcs* |
| G4Y79_21075 | Propionyl-CoA synthase | *Pcs* |
| G4Y79_13175 | Propionyl-CoA synthase | *Pcs* |
| G4Y79_18165 | Propionyl-CoA synthase | *Pcs* |
| G4Y79_19460 | Propionyl-CoA carboxylase | *Pcc* |
| G4Y79_19470 | Propionyl-CoA carboxylase | *Pcc* |
| G4Y79_22910 | Propionyl-CoA carboxylase | *Pcc* |
| G4Y79_19425 | Methylmalonyl-CoA epimerase | *Mce* |
| G4Y79_16950 | Methylmalonyl-CoA mutase | *Mcm* |
| G4Y79_16955 | Methylmalonyl-CoA mutase | *Mcm* |
| G4Y79_02905 | Succinate dehydrogenase | *Sd* |
| G4Y79_03910 | Succinate dehydrogenase | *Sd* |
| G4Y79_03915 | Succinate dehydrogenase | *Sd* |
| G4Y79_03920 | Succinate dehydrogenase | *Sd* |
| G4Y79_10075 | Succinate dehydrogenase | *Sd* |
| G4Y79_10080 | Succinate dehydrogenase | *Sd* |
| G4Y79_11585 | Succinate dehydrogenase | *Sd* |
| G4Y79_11595 | Succinate dehydrogenase | *Sd* |
| G4Y79_12425 | Succinate dehydrogenase | *Sd* |
| G4Y79_12490 | Succinate dehydrogenase | *Sd* |
| G4Y79_14625 | Succinate dehydrogenase | *Sd* |
| G4Y79_14635 | Succinate dehydrogenase | *Sd* |
| G4Y79_05870 | Fumarate hydratase | *Fh* |
| G4Y79_17370 | Fumarate hydratase | *Fh* |
| G4Y79_06165 | Mesaconyl-CoA C1-C4 CoA transferase | *Mct* |
| G4Y79_23435 | L-malyl-CoA/b-methylmaly-CoA lyase | *Mcl* |
| G4Y79_06050 | β-methylmalyl-CoA dehydratase | *Mcd* |
| G4Y79_14950 | Citramalyl-CoA lyase | *Ccl* |
| G4Y79_14970 | Citramalyl-CoA lyase | *Ccl* |
| G4Y79_03970 | Malate dehydrogenase | *Md* |
