# Supplementary material for: Characterization of the First Cultured Representative of “Candidatus Thermofonsia” Clade 2 within Chloroflexi Reveals Its Phototrophic Lifestyle
Source: mBio. 2022 Mar 1;13(2):e00287-22. doi: 10.1128/mbio.00287-22 (PMC8941918; doi:10.1128/mbio.00287-22)
Supplement: TABLE S3 [file mbio.00287-22-st003.docx]

**Supplementary Table S3.** Characteristics of the new class *Thermofonsia* (with strain ZRK33 as a representative) and other *Chloroflexi* classes. Classes: 1, *Thermofonsia* (This study); 2, *Chloroflexia* (1); 3, *Thermomicrobia* (2, 3); 4, *Anaerolineae* (4-6); 5, *Caldilineae* (4); 6, *Ardenticatenia* (7); 7, *Ktedenobacteria* (8-10); 8, *Thermoflexia* (11); 9, *Dehalococcoidetes* (12, 13). +, Positive; -, negative. This table is constructed with reference to Ward, *et al* (14).

| **Characteristic** | **1** | **2** | **3** | **4** | **5** | **6** | **7** | **8** | **9** |
| --- | --- | --- | --- | --- | --- | --- | --- | --- | --- |
| Phototrophy  Aerobic  respiration  Cell morphology  Temperature range for growth (°C)    DNA G+C content (%) | +  Microaerophilic  Filamentous    4-32  52.76 | +  +  Filamentous  10-67  48–62 | -  +  Rod  43-80  56–63 | -  Genes present  Filamentous or rods  20-73  48–58 | -  Genes present  Filamentous  37-65  59–65 | -  +  Filamentous  30-75  51.5 | -  +  Filamentous  17-74  54–60 | -  Microaerophilic  Filamentous  67.5-75  69 | -  Genes rarely present  Coccoidal, discs  15-35  49–54 |

**References related to this table**

1. Gupta RS, Chander P, George S. 2013. Phylogenetic framework and molecular signatures for the class *Chloroflexi* and its different clades; proposal for division of the class *Chloroflexia* class. nov. into the suborder *Chloroflexineae* subord. nov., consisting of the emended family *Oscillochloridaceae* and the family *Chloroflexaceae* fam. nov., and the suborder *Roseiflexineae* subord. nov., containing the family *Roseiflexaceae* fam. nov. Antonie Van Leeuwenhoek 103:99-119.

2. Hugenholtz P, Stackebrandt E. 2004. Reclassification of *Sphaerobacter thermophilus* from the subclass *Sphaerobacteridae* in the phylum *Actinobacteria* to the class *Thermomicrobia* (emended description) in the phylum *Chloroflexi* (emended description). Int J Syst Evol Microbiol 54:2049-2051.

3. Sorokin DY, Lücker S, Vejmelkova D, Kostrikina NA, Kleerebezem R, Rijpstra WI, Damsté JS, Le Paslier D, Muyzer G, Wagner M, van Loosdrecht MC, Daims H. 2012. Nitrification expanded: discovery, physiology and genomics of a nitrite-oxidizing bacterium from the phylum *Chloroflexi*. ISME J 6:2245-56.

4. Yamada T, Sekiguchi Y, Hanada S, Imachi H, Ohashi A, Harada H, Kamagata Y. 2006. *Anaerolinea thermolimosa* sp. nov., *Levilinea saccharolytica* gen. nov., sp. nov. and *Leptolinea tardivitalis* gen. nov., sp. nov., novel filamentous anaerobes, and description of the new classes *Anaerolineae* classis nov. and *Caldilineae* classis nov. in the bacterial phylum *Chloroflexi*. Int J Syst Evol Microbiol 56:1331-1340.

5. Hemp J, Ward LM, Pace LA, Fischer WW. 2015. Draft Genome Sequence of *Ornatilinea apprima* P3M-1, an Anaerobic Member of the *Chloroflexi* Class *Anaerolineae*. Genome Announc 3.

6. Pace LA, Hemp J, Ward LM, Fischer WW. 2015. Draft Genome of *Thermanaerothrix daxensis* GNS-1, a Thermophilic Facultative Anaerobe from the *Chloroflexi* Class *Anaerolineae*. Genome Announc 3.

7. Kawaichi S, Ito N, Kamikawa R, Sugawara T, Yoshida T, Sako Y. 2013. *Ardenticatena maritima* gen. nov., sp. nov., a ferric iron- and nitrate-reducing bacterium of the phylum '*Chloroflexi*' isolated from an iron-rich coastal hydrothermal field, and description of *Ardenticatenia* classis nov. Int J Syst Evol Microbiol 63:2992-3002.

8. Yabe S, Aiba Y, Sakai Y, Hazaka M, Yokota A. 2010. *Thermosporothrix hazakensis* gen. nov., sp. nov., isolated from compost, description of *Thermosporotrichaceae* fam. nov. within the class *Ktedonobacteria* Cavaletti et al. 2007 and emended description of the class *Ktedonobacteria*. Int J Syst Evol Microbiol 60:1794-1801.

9. Chang YJ, Land M, Hauser L, Chertkov O, Del Rio TG, Nolan M, Copeland A, Tice H, Cheng JF, Lucas S, Han C, Goodwin L, Pitluck S, Ivanova N, Ovchinikova G, Pati A, Chen A, Palaniappan K, Mavromatis K, Liolios K, Brettin T, Fiebig A, Rohde M, Abt B, Göker M, Detter JC, Woyke T, Bristow J, Eisen JA, Markowitz V, Hugenholtz P, Kyrpides NC, Klenk HP, Lapidus A. 2011. Non-contiguous finished genome sequence and contextual data of the filamentous soil bacterium *Ktedonobacter racemifer* type strain (SOSP1-21). Stand Genomic Sci 5:97-111.

10. Cavaletti L, Monciardini P, Bamonte R, Schumann P, Rohde M, Sosio M, Donadio S. 2006. New lineage of filamentous, spore-forming, gram-positive bacteria from soil. Appl Environ Microbiol 72:4360-9.

11. Dodsworth JA, Gevorkian J, Despujos F, Cole JK, Murugapiran SK, Ming H, Li WJ, Zhang G, Dohnalkova A, Hedlund BP. 2014. *Thermoflexus hugenholtzii* gen. nov., sp. nov., a thermophilic, microaerophilic, filamentous bacterium representing a novel class in the *Chloroflexi*, *Thermoflexia* classis nov., and description of *Thermoflexaceae* fam. nov. and *Thermoflexales* ord. nov. Int J Syst Evol Microbiol 64:2119-2127.

12. Löffler FE, Yan J, Ritalahti KM, Adrian L, Edwards EA, Konstantinidis KT, Müller JA, Fullerton H, Zinder SH, Spormann AM. 2013. *Dehalococcoides mccartyi* gen. nov., sp. nov., obligately organohalide-respiring anaerobic bacteria relevant to halogen cycling and bioremediation, belong to a novel bacterial class, *Dehalococcoidia classis* nov., order *Dehalococcoidales* ord. nov. and family *Dehalococcoidaceae* fam. nov., within the phylum *Chloroflexi*. Int J Syst Evol Microbiol 63:625-635.

13. Moe WM, Yan J, Nobre MF, da Costa MS, Rainey FA. 2009. *Dehalogenimonas lykanthroporepellens* gen. nov., sp. nov., a reductively dehalogenating bacterium isolated from chlorinated solvent-contaminated groundwater. Int J Syst Evol Microbiol 59:2692-7.

14. Ward LM, Hemp J, Shih PM, McGlynn SE, Fischer WW. 2018. Evolution of phototrophy in the *Chloroflexi* phylum driven by horizontal gene transfer. Front Microbiol 9:260.
