# Supplementary material for: Characterization of the First Cultured Representative of “Candidatus Thermofonsia” Clade 2 within Chloroflexi Reveals Its Phototrophic Lifestyle
Source: mBio. 2022 Mar 1;13(2):e00287-22. doi: 10.1128/mbio.00287-22 (PMC8941918; doi:10.1128/mbio.00287-22)
Supplement: FIG S3 [file mbio.00287-22-sf003.docx]

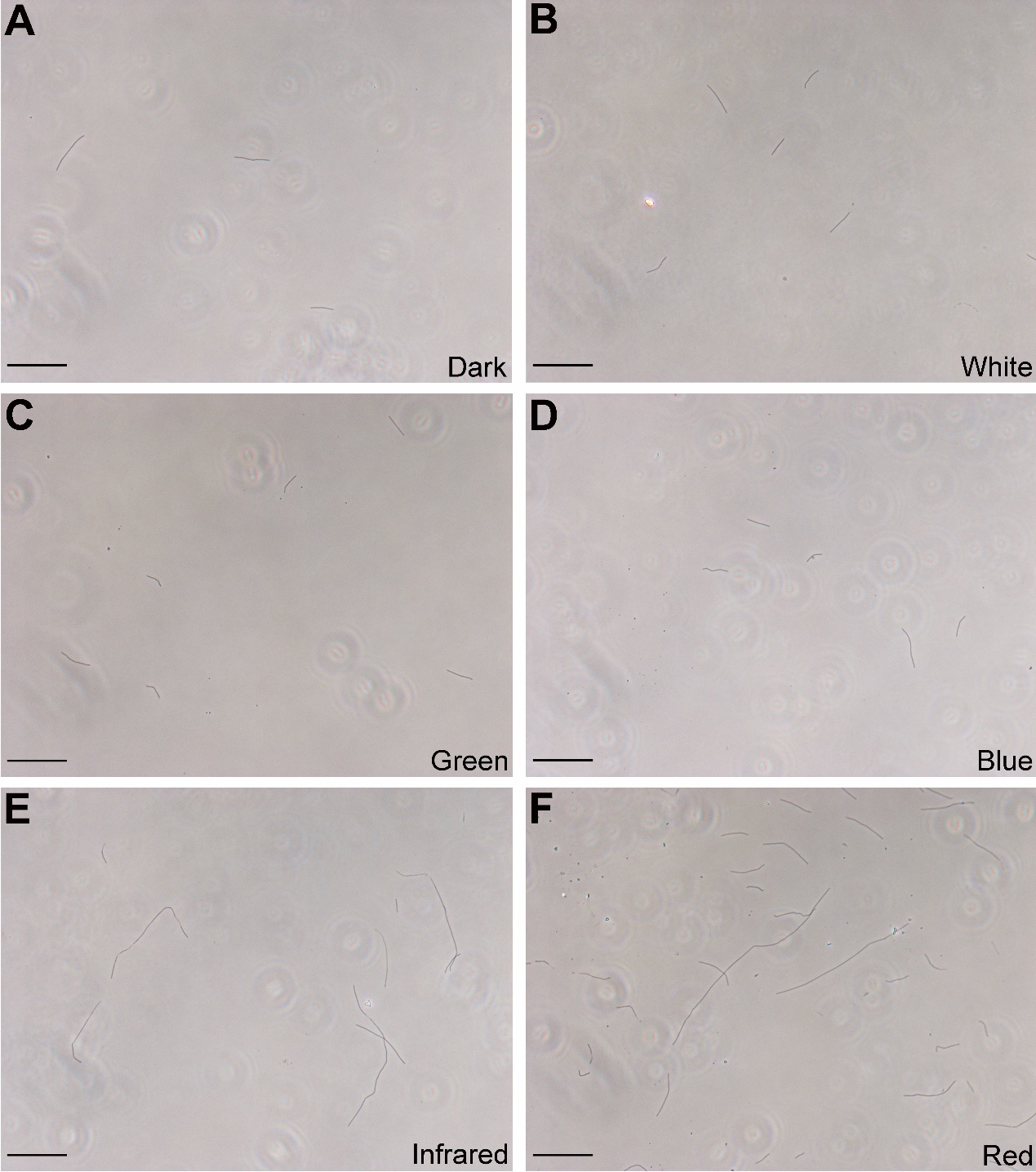


**FIG S3.** Representative pictures showing the number and length of filamentous cells of strain ZRK33 under dark (A) and different wavelengths of light illumination (including white (B), green (C), blue (D), infrared (E) and red (F)).
