# Supplementary material for: Characterization of the First Cultured Representative of “Candidatus Thermofonsia” Clade 2 within Chloroflexi Reveals Its Phototrophic Lifestyle
Source: mBio. 2022 Mar 1;13(2):e00287-22. doi: 10.1128/mbio.00287-22 (PMC8941918; doi:10.1128/mbio.00287-22)
Supplement: FIG S4 [file mbio.00287-22-sf004.docx]

**
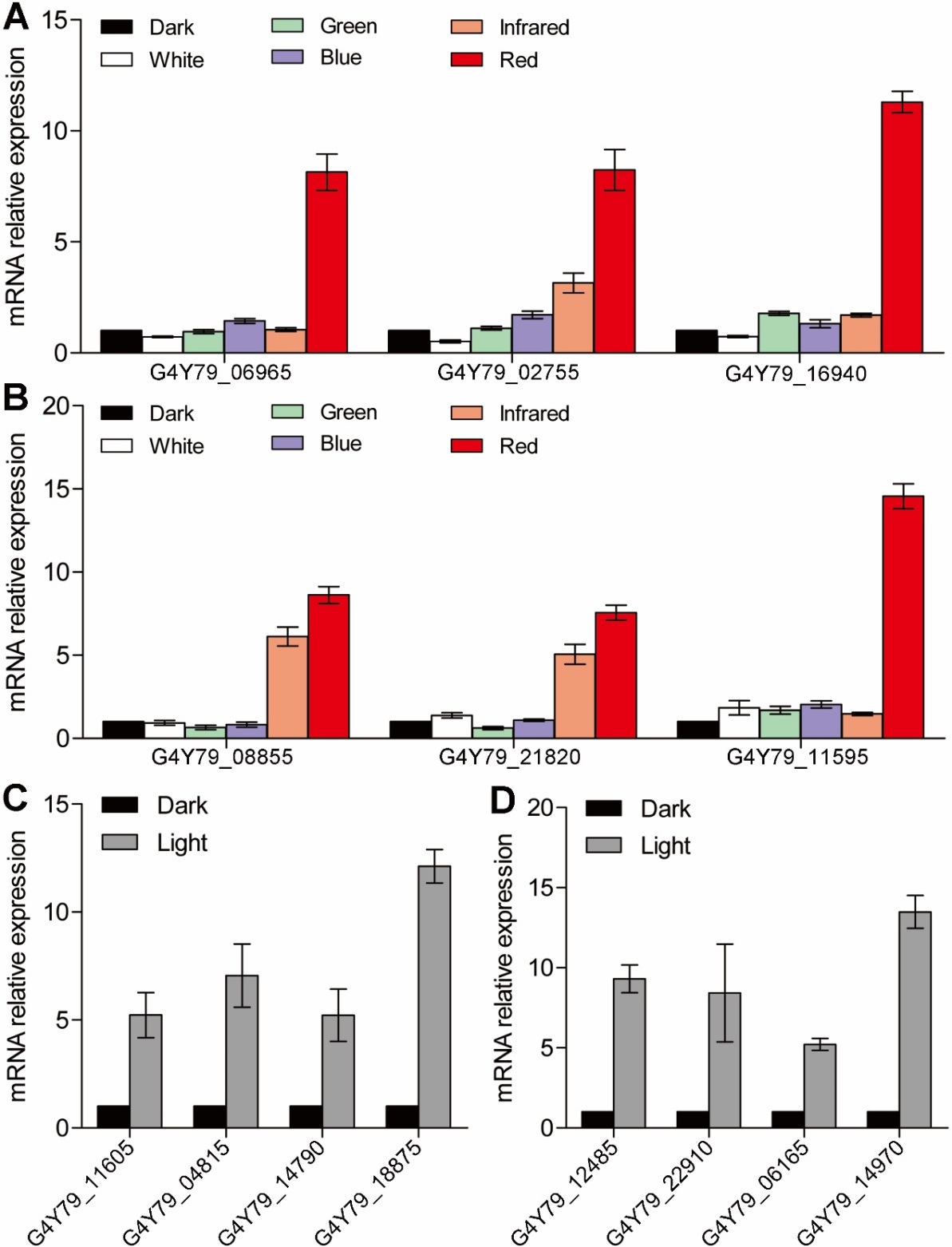
FIG S4.** qRT-PCR detection of expression changes of genes associated with phototrophic apparatus and 3HP bicycle of strain ZRK33. The expression of three genes associated with phototrophic apparatus (A) and three genes associated with 3HP bicycle (B) of strain ZRK33 exposed to darkness and different wavelengths of light illumination in the laboratory condition. The expression of three genes associated with phototrophic apparatus (C) and three genes associated with 3HP bicycle (D) of strain ZRK33 with a 10-day incubation exposed to the natural light of the deep-sea cold seep.
