# Supplementary material for: Characterization of the First Cultured Representative of “Candidatus Thermofonsia” Clade 2 within Chloroflexi Reveals Its Phototrophic Lifestyle
Source: mBio. 2022 Mar 1;13(2):e00287-22. doi: 10.1128/mbio.00287-22 (PMC8941918; doi:10.1128/mbio.00287-22)
Supplement: TABLE S4 [file mbio.00287-22-st004.docx]

**Supplementary Table S4.** The sampling sites for metagenomic analysis, and assembly statistics and quality metrics of reconstructed genome bins of *Chloroflexi* used in this study.

| **Sample name** | **Sample type** | **Location** | **Longitude and latitude** | **Depth (m)** | | |
| --- | --- | --- | --- | --- | --- | --- |
| zhu | Sediment | Cold seep | E 122º37'24.267''  N 24º57'9.016'' | 1128 | | |
| C1 | Sediment | Cold seep | E 119º17'09.106''  N 22º06'55.114'' | 1146 | | |
| C2 | Sediment | Cold seep | E 119º17'07.322''  N 22º06'58.598'' | 1121 | | |
| C4 | Sediment | Cold seep | E 119º17'06.436''  N 22º06'55.169'' | 1137 | | |
| H1 | Sediment | Hydrothermal vent | E 126º53'51.85''  N 27º47'11.26'' | 958 | | |
| H2 | Sediment | Hydrothermal vent | E 124º22'23.374''  N 25º15'49.868'' | 2194 | | |
| **Bin name** | **Taxonomy** | **Completeness (%)** | **Contamination (%)** | **GC (%)** | **N50 (bp)** | **Genome size (bp)** |
| zhu.bin.3 | Chloroflexi | 56.84 | 1.98 | 0.494 | 4097 | 748332 |
| zhu.bin.7 | Chloroflexi | 59.57 | 1.98 | 0.446 | 5817 | 588020 |
| zhu.bin.9 | Chloroflexi | 66.38 | 1.925 | 0.506 | 2239 | 1167066 |
| zhu.bin.22 | Chloroflexi | 51.94 | 8.91 | 0.52 | 6548 | 1409231 |
| zhu.bin.33 | Chloroflexi | 78.05 | 1.99 | 61.3 | 3481 | 2239783 |
| zhu.bin.44 | Chloroflexi | 66.88 | 0.99 | 0.528 | 6754 | 946329 |
| C1.bin.34 | Chloroflexi | 76.21 | 2.828 | 0.612 | 3548 | 2588152 |
| C1.bin.35 | Chloroflexi | 58.64 | 1.818 | 0.455 | 8245 | 1933721 |
| C2.bin.4 | Chloroflexi | 82.83 | 0 | 0.486 | 39431 | 941411 |
| C2.bin.6 | Chloroflexi | 70.92 | 0 | 0.495 | 7628 | 827319 |
| C2.bin.8 | Chloroflexi | 74.02 | 0.99 | 0.525 | 4817 | 757107 |
| C2.bin.9 | Chloroflexi | 80.36 | 1.98 | 0.548 | 6764 | 1051572 |
| C2.bin.12 | Chloroflexi | 54.49 | 2.727 | 0.523 | 3759 | 1643107 |
| C2.bin.17 | Chloroflexi | 65.4 | 4.158 | 0.542 | 4882 | 621181 |
| C2.bin.33 | Chloroflexi | 63.82 | 1.386 | 0.609 | 3494 | 1094429 |
| C2.bin.34 | Chloroflexi | 62.68 | 2.727 | 0.479 | 4652 | 2209326 |
| C2.bin.38 | Chloroflexi | 72.49 | 4.022 | 0.619 | 3598 | 2727830 |
| C2.bin.45 | Chloroflexi | 87.29 | 1.485 | 0.537 | 9527 | 1647588 |
| C2.bin.48 | Chloroflexi | 61.22 | 8.25 | 0.452 | 5264 | 973927 |
| C4.bin.19 | Chloroflexi | 67.43 | 0.565 | 0.644 | 3026 | 1749317 |
| H1.bin.7 | Chloroflexi | 73.68 | 0 | 0.545 | 4996 | 1405141 |
| H1.bin.32 | Chloroflexi | 71.94 | 7.727 | 0.563 | 3845 | 2766581 |
| H2.bin.45 | Chloroflexi | 76.73 | 4.378 | 0.579 | 3649 | 942250 |
| H2.bin.80 | Chloroflexi | 86.57 | 0.925 | 0.663 | 14655 | 3235763 |
| H2.bin.87 | Chloroflexi | 59.82 | 1.485 | 0.603 | 3209 | 1355795 |
| H2.bin.116 | Chloroflexi | 92.73 | 0.99 | 0.543 | 27621 | 1910274 |
| H2.bin.125 | Chloroflexi | 70.13 | 0.99 | 0.477 | 5832 | 1871708 |
