# Supplementary material for: Characterization of the First Cultured Representative of “Candidatus Thermofonsia” Clade 2 within Chloroflexi Reveals Its Phototrophic Lifestyle
Source: mBio. 2022 Mar 1;13(2):e00287-22. doi: 10.1128/mbio.00287-22 (PMC8941918; doi:10.1128/mbio.00287-22)
Supplement: TABLE S5 [file mbio.00287-22-st005.docx]

**Supplementary Table S5.** Primers used for qRT-PCR.

| **Primer name** | **Nucleotide Sequence (5’-3’)** |
| --- | --- |
| 16S-F | AGACTGGGATAACGGTCGGA |
| 16S-R | CAACTAGCTGATGGGTCGCA |
| G4Y79_06965-F | TTACGTGCCTCCCACGTTTT |
| G4Y79_06965-R | CCGGGGTGTGCTCTATTCTC |
| G4Y79_02755-F | CAAATCATTGCACGCGGGAT |
| G4Y79_02755-R | TCTCTGGTAGGGGGAGTTCG |
| G4Y79_16940-F | CCTGTACCAGTGCGGGTTAG |
| G4Y79_16940-R | GTGATGAACGAAGGCAACGG |
| G4Y79_08855-F | CTCAGCGGTGGGACAGATTT |
| G4Y79_08855-R | CGCTCTCATTGTACGCTTGC |
| G4Y79_21820-F | ATGGTCCATGCGGTTGCTAA |
| G4Y79_21820-R | AAAAAGCGCGGACCATAAGC |
| G4Y79_11595-F | TGAAGCATGCAACCTTTGGC |
| G4Y79_11595-R | GTCATTGAAGGCAGGGGTCA |
| G4Y79_11605-F | TCTCCCTGGTGAATTCGTGC |
| G4Y79_11605-R | TGCCCCGTAAACATGCTCTT |
| G4Y79_04815-F | CGAGGTGGATGGACAGATCG |
| G4Y79_04815-R | GAGTGCGGGAGTAAGCTCTC |
| G4Y79_14790-F | GGTGCCAAATGCTTTCCGTT |
| G4Y79_14790-R | ACTTCCCAACCGAGCAACAA |
| G4Y79_18875-F | CGATAGCCGCGCCAAAATAC |
| G4Y79_18875-R | TCATCTACGACGAGCGTGTG |
| G4Y79_12485-F | GAGGAAGTGCCTCTACTGCG |
| G4Y79_12485-R | ATGCCCTACTAGAAACGGCG |
| G4Y79_22910-F | AACAGTACCCGTTTCCGTCG |
| G4Y79_22910-R | GATTTTACGGCGCAGATGCC |
| G4Y79_06165-F | TTTGCAGCCGTAGCTCGTTA |
| G4Y79_06165-R | TATGGATGGGTGGGTTGTGC |
| G4Y79_14970-F | TCGACGAAATGGGCGTTGTA |
| G4Y79_14970-R | GCGATACGCGCATTCTTCAG |
